# Spermidine reduces neuroinflammation and soluble amyloid beta in an Alzheimer’s disease mouse model

**DOI:** 10.1186/s12974-022-02534-7

**Published:** 2022-07-02

**Authors:** Kiara Freitag, Nele Sterczyk, Sarah Wendlinger, Benedikt Obermayer, Julia Schulz, Vadim Farztdinov, Michael Mülleder, Markus Ralser, Judith Houtman, Lara Fleck, Caroline Braeuning, Roberto Sansevrino, Christian Hoffmann, Dragomir Milovanovic, Stephan J. Sigrist, Thomas Conrad, Dieter Beule, Frank L. Heppner, Marina Jendrach

**Affiliations:** 1 Department of Neuropathology, Charité, Universitätsmedizin Berlin, corporate member of Freie Universität Berlin, Humboldt-Universität Zu Berlin, Berlin Institute of Health, Berlin, Germany; 2 German Center for Neurodegenerative Diseases (DZNE) within the Helmholtz Association, Berlin, Germany; 3 Department of Biosciences, University of Salzburg, Salzburg, Austria; 4 Core Unit Bioinformatics, Berlin Institute of Health at Charité, Universitätsmedizin Berlin, Charitéplatz 1, Berlin, Germany; 5 Core Facility, High-Throughput Mass Spectrometry, Charité, Universitätsmedizin Berlin, corporate member of Freie Universität Berlin and Humboldt-Universität Zu Berlin, Berlin, Germany; 6 Molecular Biology of Metabolism Laboratory, The Francis Crick Institute, London, UK; 7 Department of Biochemistry, Charité, Universitätsmedizin Berlin, corporate member of Freie Universität Berlin and Humboldt-Universität Zu Berlin, Berlin, Germany; 8 Genomics Technology Platform, Max Delbrück Center for Molecular Medicine in the Helmholtz Association (MDC), Berlin, Germany; 9 Laboratory of Molecular Neuroscience, German Center for Neurodegenerative Diseases (DZNE) within the Helmholtz Association, Berlin, Germany; 10 Cluster of Excellence, NeuroCure, Berlin, Germany; 11 Institute for Biology and Genetics, Freie Universität Berlin, Berlin, Germany; 12 Berlin Institute of Health (BIH), Berlin, Germany

**Keywords:** Alzheimer’s disease, Neuroinflammation, Microglia, Astrocytes, Autophagy, Spermidine, Dietary supplement, Single nuclei sequencing, Liquid chromatography tandem mass spectrometry, Phagocytosis

## Abstract

**Background:**

Deposition of amyloid beta (Aβ) and hyperphosphorylated tau along with glial cell-mediated neuroinflammation are prominent pathogenic hallmarks of Alzheimer’s disease (AD). In recent years, impairment of autophagy has been identified as another important feature contributing to AD progression. Therefore, the potential of the autophagy activator spermidine, a small body-endogenous polyamine often used as dietary supplement, was assessed on Aβ pathology and glial cell-mediated neuroinflammation.

**Results:**

Oral treatment of the amyloid prone AD-like APPPS1 mice with spermidine reduced neurotoxic soluble Aβ and decreased AD-associated neuroinflammation. Mechanistically, single nuclei sequencing revealed AD-associated microglia to be the main target of spermidine. This microglia population was characterized by increased AXL levels and expression of genes implicated in cell migration and phagocytosis. A subsequent proteome analysis of isolated microglia confirmed the anti-inflammatory and cytoskeletal effects of spermidine in APPPS1 mice. In primary microglia and astrocytes, spermidine-induced autophagy subsequently affected TLR3- and TLR4-mediated inflammatory processes, phagocytosis of Aβ and motility. Interestingly, spermidine regulated the neuroinflammatory response of microglia beyond transcriptional control by interfering with the assembly of the inflammasome.

**Conclusions:**

Our data highlight that the autophagy activator spermidine holds the potential to enhance Aβ degradation and to counteract glia-mediated neuroinflammation in AD pathology.

**Supplementary Information:**

The online version contains supplementary material available at 10.1186/s12974-022-02534-7.

## Background

Alzheimer’s disease (AD) is the most common neurodegenerative disease and the leading cause of dementia worldwide. Pathologically, AD is defined by the following hallmarks: extracellular plaques containing amyloid-beta (Aβ), neurofibrillary tangles consisting of hyperphosphorylated microtubule-associated protein tau, loss of neurons and neuroinflammation. Over the last decade, a large body of evidence revealed a substantial involvement of microglia, the brain’s intrinsic myeloid cells, in regulating and potentially driving AD pathogenesis. Microglia are essential for maintaining brain homeostasis and respond to AD pathology by transforming into disease-associated microglia (DAM) [1], an activated and transcriptionally distinct state, which is associated with alterations in proliferation, phagocytic behavior and increased cytokine production [2]. Similarly, astrocytes produce cytokines upon activation with Aβ [3, 4]. The link between neuroinflammation and neurodegenerative diseases is strengthened by the profound effects of maternal immune activation (e.g., by poly I:C injections) on the development of neurodegenerative diseases [5–7], thus demonstrating a crucial role of inflammatory events in the initiation of a vicious cycle of neuropathological alterations.

A growing set of data, including those derived from genome-wide association studies of various human diseases by the Wellcome Trust Case Control Consortium [8], indicates that autophagy, one of the main degradation and quality control pathways of the cell, is dysregulated in AD patients and AD mouse models [9, 10]. Autophagy may interfere with AD pathology either by regulating Aβ degradation and/or by modulating neuroinflammatory processes. For both interference points, the mechanisms and target cells are still not fully understood. Mice deficient in the autophagic protein ATG16L1 exhibited a specific increase of Interleukin (IL)-1β and IL-18 in macrophages and severe colitis, which was ameliorated by anti-IL-1β and IL-18 antibody administration [11]. Recently, we could show that a reduction of the key autophagic protein Beclin1 (BECN1), which is also decreased in AD patients [12, 13], resulted in an enhanced release of IL-1β and IL-18 by microglia [14]. The multimeric NLRP3 inflammasome complex, responsible for processing Pro-IL-1β and Pro-IL-18 into its mature forms by activated Caspase-1 (CASP1) [15], was shown to be degraded by autophagy [14, 16]. Lack of the NLRP3–inflammasome axis resulted in amelioration of neuroinflammation and disease pathology in several neurodegenerative mouse models [17–19], thus emphasizing that activation of autophagy presents an intriguing therapeutic target to counteract neuroinflammation.

The small endogenous polyamine and nutritional supplement spermidine is known to induce autophagy by inhibiting different acetyltransferases [20, 21] and to extend the life span of flies, worms and yeast [21–24]. In addition, spermidine supplementation improved clinical scores and neuroinflammation in mice with experimental autoimmune encephalomyelitis (EAE) [24, 25], protected dopaminergic neurons in a Parkinson’s disease rat model [26], and exhibited neuroprotective effects and anti-inflammatory properties in a murine model of accelerated aging [27]. Consistent with these observations, spermidine decreased the inflammatory response of macrophages and the microglial cell line BV2 upon LPS stimulation in vitro [28–30]. Recent data showed that polyamines improved age-impaired cognitive function and tau-mediated memory impairment in mice [31, 32] and impaired COVID-19 virus particle production [33]. These findings led us to investigate the yet unknown potential of spermidine to interfere with AD pathology and chronic neuroinflammation.

Here, we show that spermidine treatment of the AD-like APPPS1 mice reduced soluble Aβ species. Applying single nuclei sequencing and liquid chromatography tandem mass spectrometry, crucial underlying changes in microglia, namely, the DAM marker AXL and pathways associated with cell migration, phagocytosis, autophagy and anti-neuroinflammation were identified. At later stages of disease pathology, spermidine reduced a CNS-wide AD-associated neuroinflammation in vivo*,* which correlates with targeting key inflammatory signaling pathways in vitro*.* We, therefore, provide evidence that spermidine enhances degradation of Aβ and subsequently counteracts microglia-mediated neuroinflammation.

## Materials and methods

### Mice and spermidine treatment

APPPS1^+/−^ mice [34] were used as an Alzheimer’s disease-like mouse model. *Casp1*^−/−^ mice were a kind gift from F. Knauf and M. Reichel, Medizinische Klinik m.S. Nephrologie und Internistische Intensivmedizin, Charité Berlin. Beclin1^flox/flox^ mice were a kind gift from Tony Wyss-Coray (Stanford University School of Medicine/USA).

APPPS1^+/−^ mice and littermate wild type control (WT) mice were treated with 3 mM spermidine dissolved in their drinking water (changed twice a week) from an age of 30 days until an age of either 120 days or 290 days. Control mice received only water (H_2_O). Prior to each exchange of the drinking bottles, the weight of the bottles was determined and used to calculate the average volume consumed per animal per day. Animals were kept in individually ventilated cages with a 12 h light cycle with food and water ad libitum. All animal experiments were conducted in accordance with animal welfare acts and were approved by the regional office for health and social service in Berlin (LaGeSo).

### Tissue preparation

Mice were anesthetized with isoflurane, euthanized by CO_2_ exposure and transcardially perfused with PBS. Brains were removed from the skull and sagitally divided. The left hemisphere was fixed with 4% paraformaldehyde for 24 h at 4 °C and subsequently immersed in 30% sucrose until sectioning for immunohistochemistry was performed. The right hemisphere was snap-frozen in liquid nitrogen and stored at − 80 °C for a 3-step protein extraction using buffers with increasing stringency as described previously [35]. In brief, the hemisphere was homogenized in Tris‐buffered saline (TBS) buffer (20 mM Tris, 137 mM NaCl, pH = 7.6) to extract soluble proteins, in Triton‐X buffer (TBS buffer containing 1% Triton X‐100) for membrane-bound proteins and in SDS buffer (2% SDS in ddH_2_O) for the SDS-soluble fraction of Aβ, which we here refer to as insoluble Aβ. The protein fractions were extracted by ultracentrifugation at 100,000 g for 45 min after initial homogenization with a tissue homogenizer and a 1 ml syringe with G26 cannulas. The respective supernatants were collected and frozen at − 80 °C for downstream analysis. Protein concentration was determined using the Quantipro BCA Protein Assay Kit (Pierce) according to the manufacturer’s protocol with a Tecan Infinite^®^ 200 Pro (Tecan Life Sciences).

### Quantification of Aβ levels and pro-inflammatory cytokines

Aβ40 and Aβ42 levels of brain protein fractions were measured using the 96‐well MultiSpot V-PLEX Aβ Peptide Panel 1 (6E10) Kit (MesoScale Discovery, K15200E-1). While the TBS and TX fraction were not diluted, the SDS fraction was diluted 1:500 with Diluent 35. Cytokine concentrations were measured in the undiluted TBS fraction or in the cell supernatant using the V‐PLEX Pro‐inflammatory Panel 1 (MesoScale Discovery, K15048D1). For all samples, duplicates were measured and concentrations in the TBS fraction normalized to BCA values.

### Histology

Paraformaldehyde-fixed and sucrose-treated hemispheres were frozen and cryosectioned coronally at 40 µm using a cryostat (Thermo Scientific HM 560). Details for the different staining procedures are described in the Additional file 2.

### Brain slice culture

The brains of C57Bl/6J and APPPS1 mice were harvested, the cerebellum removed and the hemispheres mounted on a cutting disk using a thin layer of superglue. Hemispheres were cut using the Vibratome platform submerged in chilled medium consisting of DMEM medium (Invitrogen, 41966-029) supplemented with 1% penicillin/streptomycin (Sigma, P0781-20ML). Coronal slicing was performed from anterior to posterior after discarding the first 1 mm of tissue generating 10 × 300 µm sequential slices per brain with vibrating frequency set to 10 and speed to 3. Brain slices were cultured in pairs in 1 ml culture medium at 35 °C, 5% CO_2_ in 6-well plates. Pre-treatment with the indicated spermidine concentrations was started immediately for 2 h. Subsequently, LPS (10 µg/ml) was added to the medium for 3 h followed by the addition of ATP (5 mM) for an additional 3 h. Afterwards, the culture medium was frozen for subsequent analyses.

### Isolation and culture of adult microglia

Adult microglia were isolated from the hemispheres of 160-day-old C57BL/6 J mice by magnetic activated cell sorting (MACS). The manufacturer’s protocols were followed. In brief, mice were transcardially perfused with PBS and tissue dissociated with the Neural Tissue Dissociation kit (P) (Miltenyi Biotec, 130‐092‐628) in C-tubes (Miltenyi, 130-096-334) on a gentleMACS Octo Dissociator with Heaters (Miltenyi Biotec, 130‐096‐427). Afterwards, the cell suspension was labelled with CD11b microbeads (Miltenyi Biotec, 130‐093‐634) and passed through LS columns (Miltenyi Biotec, 130‐042‐401) placed on an OctoMACS™ manual separator. Subsequently, microglia were collected by column flushing and cultured in DMEM medium (Invitrogen, 41966-029) supplemented with 10% FBS (PAN-Biotech, P40-37500) and 1% penicillin/streptomycin (Sigma, P0781-20ML). Medium was changed every 3 days until adult microglia were treated as indicated after 8 days in vitro (DIV).

### Cell culture of neonatal microglia and astrocytes

Newborn mice (1–4 days) were sacrificed by decapitation. Mixed glial cultures were prepared as described previously [14]. In brief, brains were dissected, meninges removed and brains mechanically and enzymatically homogenized with 0.005% trypsin/EDTA. Cells were cultured in complete medium consisting of DMEM medium (Invitrogen, 41966-029) supplemented with 10% FBS (PAN-Biotech, P40-37500) and 1% penicillin/streptomycin (Sigma, P0781-20ML) at 37 °C with 5% CO_2_. From 7 DIV on, microglia proliferation was induced by adding 5 ng/ml GM-CSF (Miltenyi Biotec, 130‐095‐746) to the complete medium. Microglia were harvested at 10–13 DIV by manually shaking flasks for 6 min. Cells were treated after a settling time of 24 h. Neonatal BECN1^flox/flox^·CX3CR1^CreERT2^ microglia were treated with (Z)-4-Hydroxytamoxifen (Sigma #7904) after 5 DIV and assessed 7 days after Tamoxifen treatment.

After isolating neonatal microglia, neonatal astrocytes were separated by MACS. Neonatal astrocytes were detached with 0.05% trypsin, pelleted by centrifugation and incubated with CD11b microbeads (Miltenyi Biotec, 130‐093‐634) for 15 min at 4 °C to negatively isolate astrocytes. Afterwards, the cell suspension was passed through LS columns (Miltenyi Biotec, 130‐042‐401) placed on an OctoMACS™ manual separator and the flow-through containing the astrocytes was collected. Subsequently, astrocytes were cultured in complete medium for 2 days before being treated. For all experiments, 100,000 cells were seeded on 24 well plates if not stated otherwise.

### Cell treatment

For pro-inflammatory stimulation, cells were either treated with LPS (1 μg/ml, Sigma, L4391-1MG) for 3 h followed by ATP (2 mM, Sigma Aldrich, A6419-5 g; 4 mM for ASC speck/inflammasome formation) for 45 min, with poly I:C (50 µg/ml, InVivoGen, tlrl-picw-250) for 6 h or with oligomeric Aβ (5 µM, Cayman Chemicals) for 24 h if not stated otherwise. Spermidine trihydrochloride (Sigma, S2501-5G) diluted in complete medium was added as indicated. For the ARPC3 western blot, cells were pretreated with spermidine for 6 h and subsequently stimulated with LPS (1 μg/ml) for 6 h followed by ATP (2 mM) for 45 min. Autophagy was activated by keeping cells in HBSS for 2 h prior to treatment (24020-091, Invitrogen) and blocked by addition of 3-MA (Sigma-Aldrich, M9282, final concentration 10 mM). The ASC oligomerization inhibitor MCC950 (inh-mcc, Invivogen) was used with a final concentration of 300 nM.

### Cell migration/chemotaxis assay

Cell migration was assessed using the Cell Migration/Chemotaxis Assay Kit (96-well, 8 µm) (ab235673). The manufacturer’s instructions were followed, and a standard of dyed cells was prepared for each biological replicate. Cell numbers were proportional to the fluorescence at Ex/Em = 530/590 measured with an Infinite® 200 Pro (Tecan Life Sciences) plate reader. As migration inducing stimulus ATP (300 µM, Sigma Aldrich, A6419-5 g) was used in the bottom chamber. Cells were seeded at a density of 50,000 cells per well in the top chamber and if treated, supplemented with 10 µM spermidine trihydrochloride (Sigma, S2501-5G). In both chambers DMEM medium (Invitrogen, 41966-029) supplemented with 1% penicillin/streptomycin (Sigma, P0781-20ML) was used. After an incubation of approximately 20 h at 37 °C with 5% CO_2_ the cells remaining on top of the membrane were removed with a cotton swab and cells that adhered on the bottom were dissociated. The number of cells migrated through the semipermeable membrane of the Boyden chamber was calculated based on the measured fluorescence and the generated linear regression standard curve with a range of 0–12,500 cells.

### Wound healing/scratch assay

Cells were seeded at a density of 300,000 cells/well of a 24-well plate. After 8 h of adherence, cells were treated with 3 µM or 10 µM spermidine trihydrochloride (Sigma, S2501-5G) in complete medium. After 15 h of incubation, the cell layer was scratched with a 200 µl pipette tip. Images were taken with a Zeiss Axio Observer Z1 Inverted Phase Contrast Fluorescence Microscope using the Zen 2 blue software for 72 h at the indicated timepoints. Acquired images were analyzed using ImageJ by defining the gap area right after scratching (0 h) as region of interest. The threshold was set to include every cell inside the region of interest, the area fraction in percent was measured and normalized to the respective value at 0 h.

### Western blot

For ASC crosslinking, 1 mM DSS (Thermo, A39267) was added to freshly harvested microglia in PBS for 30 min. All cell pellets were lysed in protein sample buffer containing 0.12 M Tris–HCl (pH 6.8), 4% SDS, 20% glycerol, 5% β-mercaptoethanol. Proteins were separated by Tris-Tricine polyacrylamide gel electrophoresis (PAGE) and transferred by wet blotting onto a nitrocellulose membrane. After blocking with 1% skim milk in Tris-buffered saline with 0.5% Tween20 (TBST), primary antibodies were added (Additional file 2). For signal detection the SuperSignal West Femto Maximum Sensitivity Substrate (ThermoFisher, 34096) was used. Western blots were analyzed by quantifying the respective intensities of each band using ImageJ. All samples were normalized to ACTIN levels or whole protein content in the supernatant.

### Quantitative real‐time PCR

For total RNA isolation, the RNeasy Mini kit (Qiagen, 74104) was used and cells were directly lysed in the provided RLT lysis buffer in the cell culture plate. Reverse transcription into cDNA was performed using the High-Capacity cDNA Reverse Transcription kit (ThermoFisher, 4368813). The manufacturer’s instructions for both kits were followed. Quantitative PCR was conducted on a QuantStudio 6 Flex Real‐Time PCR System (Applied Biosystems) using 12 ng cDNA per reaction. Gene expression was analyzed in 384 well plates using the TaqMan Fast Universal Master Mix (Applied Biosystems, 4364103) and TaqMan primers as described in the Additional file 2. With the Double delta Ct method, values were normalized to the house keeping gene *Actin* and non-treated controls.

### ELISA

Cytokine concentrations in the supernatant of cultured cells were measured using the IL‐1β (eBioscience, 88701388), IL-18 (Thermo Fisher, 88-50618-22), TNF-α (eBioscience, 88723477) and IL‐6 (eBioscience, 88706488) enzyme‐linked immunosorbent assay (ELISA) kit according to manufacturer´s instructions. The absorption was read at a wavelength of 450 nm and a reference length of 570 nm with the microplate reader Infinite® 200 Pro (Tecan Life Sciences) and analyzed using the Magellan Tecan Software.

### Immunocytochemistry and confocal microscopy

Cells were seeded at a density of 50,000 cells per well on 12 mm coverslips. After treatment, cells were fixed with 4% paraformaldehyde for 20 min, permeabilized with 0.1% Triton X-100 in PBS for another 20 min and blocked with 3% bovine serum in PBS for 1 h. The primary antibodies (anti-ASC, AdipoGen, AG-25B-0006, 1:500; anti-IBA1, Wako 019-19741, 1:1000) were added overnight at 4 °C. Subsequently, cells were incubated with the fluorescent secondary antibodies (Alexa Fluor 568-conjugated anti-rabbit IgG, 1:500, Invitrogen, A11011; 488-conjugated anti-rabbit IgG, Invitrogen A21206) for 3 h at room temperature. Cell nuclei were counterstained with DAPI (Roche, 10236276001) and coverslips embedded in fluorescent mounting medium (Dako, S3023). Images were acquired using Leica TCS SP5 confocal laser scanning microscope controlled by LAS AF scan software (Leica Microsystems, Wetzlar, Germany). Z-stacks were taken and images presented as the maximum projection of the z-stack. The number and size of ASC specks was assessed using ImageJ software as described before [14].

### Aβ preparation

*Labeling.* Aβ 1–42 peptides (Cayman Chemicals) were resuspended in hexafluoroisopropanol to obtain 1 mM solution, evaporated and stored as aliquots. For each preparation, 125 µg of amyloid-β was dissolved in 2 µL DMSO, sonicated for 10 min in the waterbath and supplemented with 3 × molar excess of NHS-ester ATTO647N dye (Sigma) in 1 × PBS (phosphate buffer saline, Gibco) and pH was adjusted to 9 with sodium bicarbonate. After 1 h of labeling reaction in the dark at room temperature, the labeled peptides were separated using spin columns (Mobicol, Mobitec) and loaded with 0.7 mL of Sephadex G25 beads (Cytiva). Clean, chromatography-grade H_2_O (LiChrosolv LC–MS grade, Merck) was used for washing, equilibration and elution. Peptide concentrations were determined using 15% SDS–PAGE gels and comparing the band intensities of the input with the eluted fractions.

*Maturation.* Aβ peptides were matured according to [36]. In short, to obtain oligomeric forms, Aβ was resuspended in the final concentration of 1 × PBS and incubated at 4 °C overnight. Turbidity measurements and ThT aggregation assay were performed on Synergy H1 Hybrid Multi-Mode Microplate Reader (BioTek instruments) to determine the formation of Aβ oligomers and fibrils, as described in [37].

### Phagocytosis assay

Neonatal microglia (50,000 cells/ 24 well) were seeded on coverslips and pre-treated for 18 h with spermidine. 0.5 µM 647-labelled Aβ was added and after 24 h cells were fixed and counterstained with anti-IBA1 (Wako 019-19741, 1:1000). Quantification of Z-stacks taken at the confocal microscope with constant settings was performed with Image J: a mask was created for each IBA1 stained cell body and the intensity of the Aβ signal in every cell was determined. The mean intensity/phagocytic cell was calculated as well as the number of Aβ-containing phagocytic cells.

### Single nuclei sequencing (snRNA-seq)

Nuclei preparation, single nuclei sequencing and single nuclei sequencing analysis are described in the Additional file 2. The dataset has been deposited in the GEO database, GSE206202.

### Proteomics analysis

Sample preparation, Liquid chromatography mass spectrometry and data analysis are described in the Additional file 2. The mass spectrometry proteomics data have been deposited with the ProteomeXchange Consortium via the PRIDE partner repository with the dataset identifier PXD034638.

### Data analysis

All values are presented as mean ± SEM (standard error of the mean). All data sets were tested for normality using the Shapiro–Wilk test. For normally distributed data, parametric tests were used: the student’s *t* test for pairwise comparisons or a one-way ANOVA using the indicated post hoc test for multiple comparisons. If the data distribution was not normal, the corresponding non-parametric tests Mann–Whitney *U* test or Kruskal–Wallis test with Dunn’s multiple comparison test were applied. As a reference for the Dunnett’s post hoc test or the Dunn’s multiple comparison either LPS/ATP, poly I:C or Aβ samples were used. Outlier testing was performed using the ROUT method (Q = 0.5%). Statistically significant values were determined using the GraphPad Prism software and are indicated as follows: *p < 0.05, **p < 0.01 and ***p < 0.001.

## Results

### Spermidine reduced soluble Aβ in APPPS1 mice

**Fig. 1:**
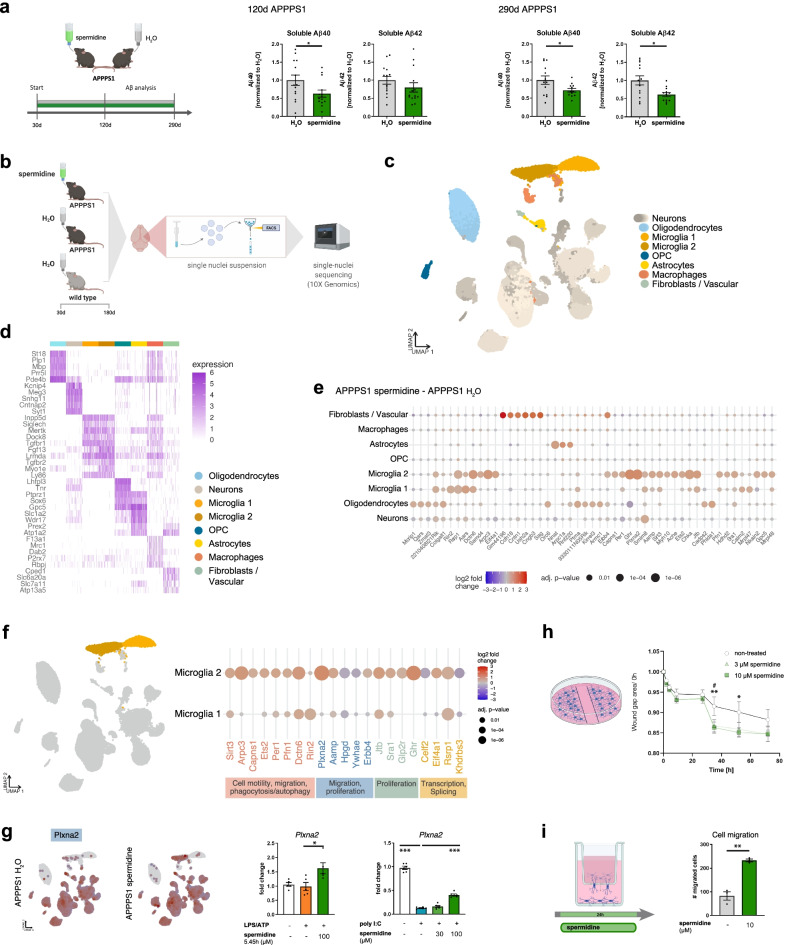
Spermidine reduced soluble Aβ and induced transcriptomic alterations in microglia of APPPS1 mice. **a** APPPS1 mice were treated with 3 mM spermidine via their drinking water starting at 30 days (d) until mice reached an age of 120 days or 290 days according to the depicted treatment scheme. Spermidine-treated APPPS1 mice were compared to non-treated controls (H_2_O). The Aβ40 and Aβ42 content was measured in the TBS (soluble) fraction of brain homogenates of 120-day-old or 290-day-old spermidine-treated mice and water controls (mixed sex) using electrochemiluminescence (MesoScale Discovery panel). Values were normalized to water controls. 120d APPPS1 H_2_O (*n* = 14), 120d APPPS1 spermidine (*n* = 14), 290d APPPS1 H_2_O (*n* = 14), 290d APPPS1 spermidine (*n* = 12); two‐tailed *t*‐test, Aβ42 in 120d mice: Mann–Whitney *U* test. **b** Single nuclei sequencing of hemispheres harvested from 180-day-old male spermidine-treated APPPS1, H_2_O APPPS1 and H_2_O control mice was performed of FACS-sorted DAPI-stained nuclei using the 10x Genomics platform (*n* = 3). **c** UMAP embedding and clustering of the snRNA-seq data, together with annotation of the major cell types. **d** Heatmap showing the top 5 marker genes for 300 cells in each of the major cell types. **e** Dot plot for the top 50 genes in a cell-type-specific differential expression analysis between spermidine-treated APPPS1 and H_2_O APPPS1 mice. Color scale indicates log2 fold change, dot size indicates adjusted *p* value. **f** Same as e, for selected genes differentially expressed in microglia clusters 1 or 2. Associated pathways are color-coded. **g** Expression of *Plxna2* in APPPS1 spermidine and APPPS1 H_2_O mice. Color scale indicates normalized expression, grey dots represent no data (left panels). For validation, neonatal microglia were treated with the indicated concentrations of spermidine in combination with LPS (1 µg/ml) and ATP (2 mM) or with poly I:C (50 µg/ml) and the gene expression was assessed by RT-qPCR (right panels). *Plxna2* expression was normalized to *Actin* and displayed as fold change compared to non-treated control cells; *n* = 5–6, one-way ANOVA, Dunnett’s post hoc test. **h** Neonatal microglia were pre-treated with 3 or 10 µM spermidine for 15 h. The confluent cell layer was scratched and the scratch area was imaged for 72 h at the indicated timepoints. The gap area normalized to timepoint 0 h is displayed; *n* = 5–6, two-way ANOVA, Dunnett’s post hoc test. **i** Neonatal microglia were non-treated or treated with 10 µM spermidine and their migration towards 300 µM ATP was quantified after 24 h using a transwell migration assay; two‐tailed *t*‐test. *p < 0.05, **p < 0.01, ***p < 0.001

To assess the potential of spermidine on AD pathology, we investigated its effects on APPPS1 mice. This AD-like mouse model, which harbors transgenes for the human amyloid precursor protein (APP) bearing the Swedish mutation as well as a presenilin 1 mutation, develops a strong Aβ pathology including neuroinflammation. APPPS1 mice were treated with 3 mM spermidine via their drinking water [31], starting prior to disease onset (namely, substantial Aβ deposition), at the age of 30 days (Fig. 1a). Compared to control APPPS1 mice that received pure water (H_2_O), spermidine-supplemented animals showed no differences in fluid uptake per day (Additional file 1: Fig. S1a).

Aβ deposition was analyzed at an intermediate disease state (120 days) and at 290 days, when pathology is known to have reached a plateau. After consecutive protein extractions, soluble and insoluble/SDS soluble Aβ40 and Aβ42 were measured by electrochemiluminescence (MesoScale Discovery panel). Spermidine supplementation significantly reduced soluble Aβ40 in both 120- and 290-day-old APPPS1 mice by 40% and soluble Aβ42 in 290-day-old mice by 49% (Fig. 1a) while not affecting insoluble Aβ (Additional file 1: Fig. S1b). These findings were further substantiated by the fact that no differences in Aβ plaque covered area or plaque size were observed after staining tissue sections with the fluorescent dye pFTAA (Additional file 1: Fig. S1c). Mechanistically, spermidine treatment did neither affect APP production and cleavage nor BACE1 abundance in whole hemisphere lysates or in proximity to 4G8-positive Aβ plaques (Additional file 1: Fig. S1d–f). As the Aβ-degrading enzyme IDE (insulin-degrading enzyme) was also not altered by spermidine treatment (Additional file 1: Fig. S1g), we concluded that spermidine might target soluble Aβ by altering its phagocytosis and/or degradation.

### Spermidine treatment of APPPS1 mice induced transcriptomic alterations in microglia

To gain insights into the molecular mechanisms mediating the reduced soluble Aβ levels and the cell populations affected by spermidine, comparative single nuclei sequencing (snRNA-seq) was performed. Hemispheres of three male spermidine-treated APPPS1 mice, H_2_O-treated APPPS1 control as well as wild type (WT) mice were analyzed at the age of 180 days, representing a midpoint in the course of pathology in this AD-like mouse model (Fig. 1b). Using fluorescence-activated cell sorted single nuclei and the 10x Genomics platform (Additional file 1: Fig. S2a), between 6500 and 10,000 cells per mouse at a median depth of 1400–1700 genes could be detected. Automated clustering revealed 34 clusters, which were grouped into 7 major cell types, namely, neurons, oligodendrocytes, microglia, oligodendrocyte precursors (OPC), astrocytes, macrophages and fibroblasts/ vascular cells, using label transfer from a previously published mouse brain reference data set [38] (Fig. 1c, d).

Interestingly, the strongest transcriptional changes were found in microglia. Fewer genes were altered in oligodendrocytes, neurons, and astrocytes, while OPC and macrophages remained almost unaffected (Fig. 1e, Additional file 1: Fig. S2f). In agreement with previous single cell transcriptomic analyses of APPPS1 mice [1], two microglia subpopulations were detected. The microglia 2 cluster appeared only in APPPS1 but not in WT mice, thus presenting an AD-associated activated microglia phenotype, which was largely equivalent to the classical DAM published by Keren-Shaul et al. [1] (Additional file 1: Fig. S2b–d). To discover the main characteristics of both microglia clusters, differential gene expression followed by gene set enrichment analysis between these populations was performed. Compared to cluster 1, the AD-associated cluster 2 revealed a downregulation of genes involved in phagocytosis, endocytosis, cell adhesion and cell polarity while upregulating neuroinflammatory responses, cell-cycle transition and autophagy (Additional file 1: Fig. S2e).

Next, genes differentially expressed in spermidine-treated APPPS1 mice compared to H_2_O-treated controls were specifically assessed in microglia clusters 1 and 2. Among the top differentially expressed genes in microglia were genes associated with cell motility/ cell migration (*Arpc3, Capns1, Pfn1, Plxna2, Aamp, Erbb4, Ywhae, Hpgd*), phagocytosis (*Arpc3, Capns1, Pfn1, Dctn6, Rin2*), autophagy (*Arpc3, Capns1, Ets2, Per1, Ghr*), proliferation (*Aamp, Ets2, Erbb4, Hpgd, Glp2r, Ghr, Jtb, Sra1, Ywhae*), transcription and alternative splicing (*Celf2, Eif4a1, Rsrp1, Khdrbs3*) (Fig. 1f; Additional file 1: Fig. S2g). Gene set enrichment analysis correspondingly revealed the following Gene Ontology terms to be significantly regulated by spermidine: glial cell migration, microtubule organization center localization, cell matrix adhesion and the semaphorin–plexin signaling pathway (Additional file 3).

To validate these changes, neonatal microglia were isolated and either activated with LPS followed by ATP, inducing the TLR4 pathway, or with the viral dsRNA poly I:C stimulating the TLR3 pathway (Fig. 4b, c). Indeed, spermidine treatment upregulated Plexin A2 (*Plxna2)* expression of activated microglia (Fig. 1g). On the functional level, spermidine treatment of neonatal microglia increased the migration in a scratch wound healing assay (Fig. 1h, Additional file 1: Fig. S2h) and towards ATP in a transwell migration assay (Fig. 1i), correlating well with the snRNA-seq changes of cell migration genes. Furthermore, spermidine augmented the expression of the autophagy-associated gene *Ets2 *in vitro (Additional file 1: Fig. S2i). Also, distinct anti-inflammatory-associated genes, *Pfn1* [39], *Glp2r* [40], *Per1* [41] and *Sirt3* [42, 43], were upregulated by spermidine in the AD-associated microglia cluster 2*.* This upregulation of the anti-inflammatory NAD-dependent deacetylase *Sirt3* was confirmed in activated neonatal microglia in vitro (Additional file 1: Fig. S2j).

We, therefore, hypothesize that spermidine prolongs and expands the early activated state of microglia, characterized by increased phagocytosis, cell motility, migration and proliferation, thus maintaining the surveillance mode of microglia and thereby reducing soluble Aβ.

### Spermidine altered AD-associated microglia and their capacity to degrade Aβ

**Fig. 2:**
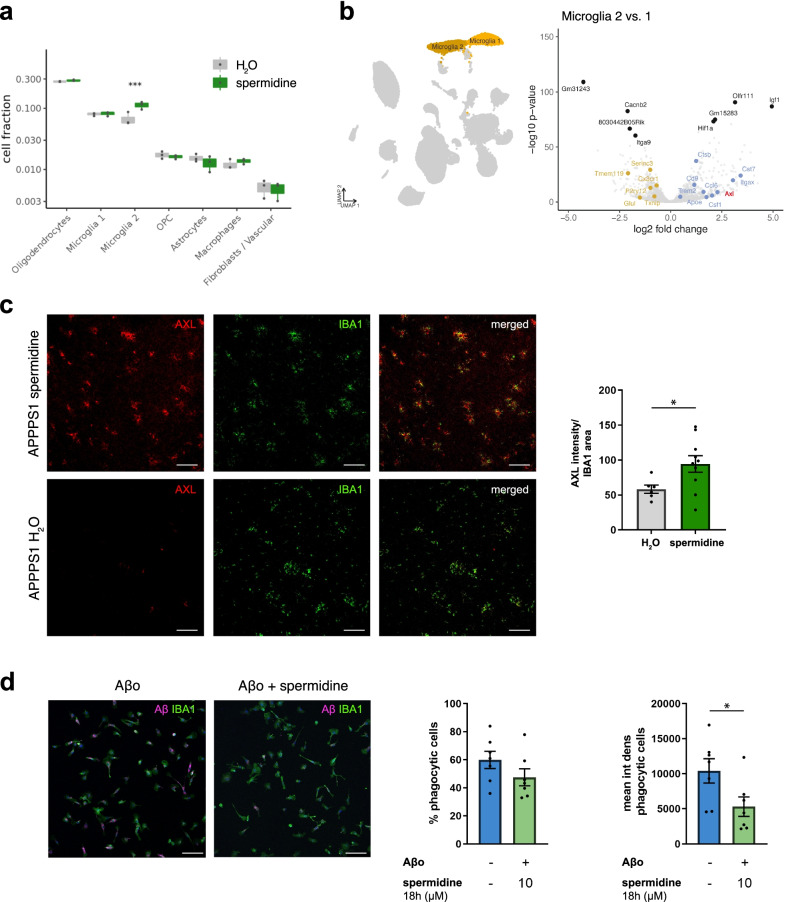
Spermidine altered AD-associated microglia and their capacity to degrade Aβ. **a** Cluster abundance in snRNA-seq of male spermidine-treated APPPS1 and H_2_O APPPS1 mice with *p* values from mixed-effects binomial model. **b** Volcano plot of genes differentially expressed in microglia cluster 2 vs. 1. The top 5 up- and down-regulated genes are indicated as well as previously published markers for homeostatic (yellow) and disease-associated (blue) microglia [1]. Significance threshold of adj. *p* value < 0.01 was used. *Axl* as a gene of interest is highlighted in red. **c** Tissue sections of male 180-day-old mice were stained for the microglia cluster 2 marker AXL (red) and IBA1 (green). The AXL intensity normalized to the IBA1 area was determined by ImageJ analysis; *n* = 6–10, two‐tailed *t*‐test. **d** Neonatal microglia were pre-treated for 18 h with 10 µM spermidine and fluorescently labelled oligomeric (Aβo) Aβ (magenta) was added for further 24 h. Microglia cells were stained for IBA1 (green). The percentage of phagocytic cells and the Aβ mean intensity density per phagocytic cell was assessed by confocal microscopy. Representative images are shown; *n* = 7, two‐tailed *t*‐test. *p < 0.05, **p < 0.01, ***p < 0.001

Next, the abundance of cell types was compared between spermidine and H_2_O APPPS1 mice. Interestingly, spermidine significantly increased the abundance of microglia cluster 2 (Fig. 2a), which correlates well with the induction of proliferation-associated genes by spermidine, and reduced levels of the anti-proliferatory gene *Hpgd* after acute spermidine treatment in vitro (Additional file 1: Fig. S2k).

To validate that spermidine indeed altered the DAM/microglia cluster 2, APPPS1 mice treated with spermidine were stained for the established cluster 2 marker and receptor tyrosine kinase AXL (Fig. 2b). In line with the snRNA-seq, the AXL intensity normalized to the IBA1 area was significantly increased in spermidine-treated mice (Fig. 2c). As the AXL-GAS6 signaling pathway was shown to promote phagocytosis and reduce Aβ load [44], the effect of spermidine on phagocytosis of Aβ was assessed in vitro*.* Spermidine pre-treatment of neonatal microglia significantly decreased the mean Aβ signal per phagocytic cell after 24 h, indicating enhanced Aβ degradation, while the percentage of phagocytic cells was not altered (Fig. 2d). In line with this, spermidine treatment increased the expression of the phagocytosis-associated actin nucleation gene *Arpc3* in APPPS1 mice*,* which could be validated on mRNA and protein level in spermidine-treated neonatal microglia in vitro (Additional file 1: Fig. S2l). Accordingly, spermidine reduced the expression of the transcriptional regulator *Celf2* (Fig. 1f), which negatively regulates the phagocytic receptor TREM2 [45] and preserved the levels of *Trem2* in activated neonatal microglia in vitro (Additional file 1: Fig. S2m).

Correlating well with the observed reduction in soluble Aβ in spermidine-treated APPPS1 mice, these results show that spermidine indeed alters phagocytosis and degradation of Aβ.

### Spermidine treatment reduced progressive neuroinflammation in APPPS1 mice

**Fig. 3:**
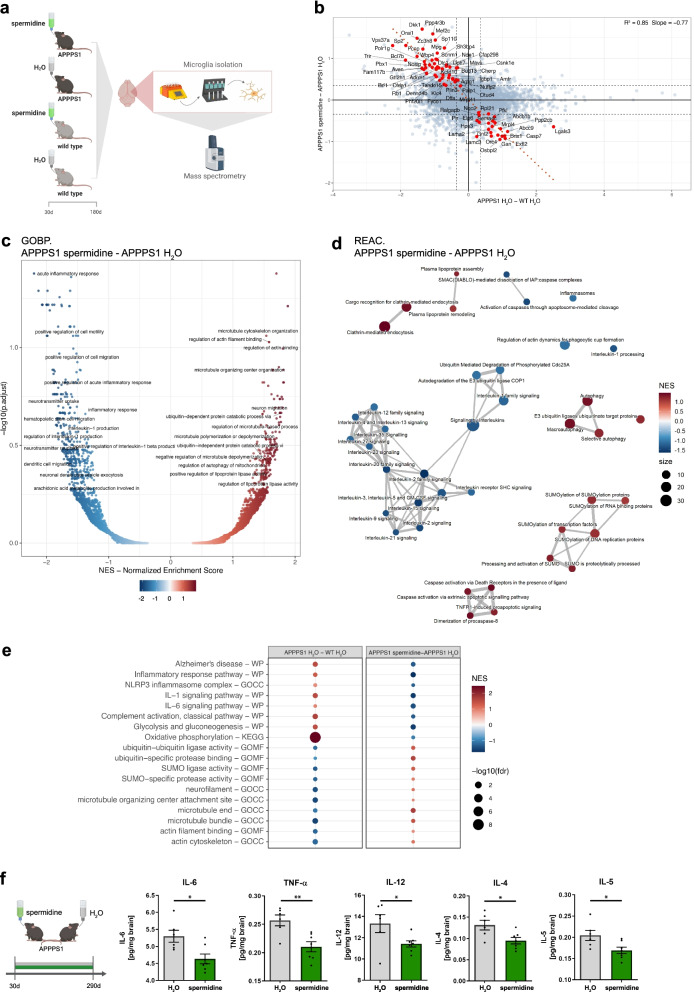
Spermidine treatment reduced progressive neuroinflammation in APPPS1 mice. **a** Male APPPS1 mice were treated with 3 mM spermidine via their drinking water starting at 30 days (d) until mice reached an age of 180 days. Microglia were isolated by MACS and the proteome assessed by mass spectrometry. **b** Scatterplot of protein regulation in Contrast2 (APPPS1 spermidine—APPPS1 H_2_O, *y* axis) vs. its regulation in Contrast1 (APPPS1 H_2_O,—WT H_2_O, *x* axis). Contrast 2 shows regulation due to spermidine effect, Contrast 1 shows the regulation of proteins by Alzheimer disease. Proteins that are regulated by spermidine and show significant anti-APPPS1 effect were marked in red. As such we selected proteins that show significant (alpha = 0.04) regulation due to spermidine in APPPS1 mice (Contrast2) and simultaneously, show significant (alpha = 0.04) effect in Contrast5 = (Contrast2 − Contrast1)/2, in the direction, opposite to the effect of the AD-like model. **c** Volcano plot of GSEA enrichment of GO BP terms. *x*-axis shows normalized enrichment score of functional term, *y*-axis represent the − log10 of its false discovery rate. Labelled are only terms that have relation to neurodegeneration and inflammation. As such we selected terms that have in their names following strings: neuro, inflamm, Clathrin, interleukin, Caspase, TNF, ubiquitin, SUMO, Alzheimer, Parkinson, Huntington, lipoprotein, autophagy, cell migration, cell motility, microtubule, actin, actin-, glia, amyloid. Not all labels appear due to strong overlap, especially at high fdr > ~ 0.5 (− log10(fdr) < ~ 0.3). Long term names are truncated to 50 characters. **d** GSEA enrichment map using top 50 REACTOME terms from list of neurodegeneration and inflammation terms. As such we selected terms that have in their names following strings: neuro, inflamm, Clathrin, interleukin, Caspase, TNF, ubiquitin, SUMO, Alzheimer, Parkinson, Huntington, lipoprotein, autophagy, cell migration, cell motility, microtubule, actin, actin-, glia, amyloid. **e** Dot plot of selected functional terms related to neuroinflammation and degeneration. **f** APPPS1 mice were treated with 3 mM spermidine via their drinking water starting at 30 days until mice reached an age of 290 days. The content of the indicated pro-inflammatory cytokines was measured in the TBS (soluble) fraction of brain homogenates of male spermidine-treated mice and water controls using electrochemiluminescence (MesoScale Discovery panel). Values were normalized to water controls. 290d APPPS1 H_2_O (*n* = 14), 290d APPPS1 spermidine (*n* = 12); two‐tailed *t*‐test. *p < 0.05, **p < 0.01, ***p < 0.01

Aβ pathology and its associated changes in microglia are a generally accepted and crucial driver of neuroinflammation [2, 8]. To complement the observed transcriptomic changes, we performed an unbiased proteome screening using liquid chromatography tandem mass spectrometry of microglia isolated from 180-day-old APPPS1 mice treated with spermidine or H_2_O as well as WT controls (Fig. 3a). To reveal the effect of spermidine on AD pathology, we applied linear modelling integrating the information of proteins changing upon spermidine treatment of APPPS1 as well as between WT and APPPS1 mice [(APPPS1 spermidine + WT H_2_O)/2 − APPPS1 H_2_O)]. The 72 differentially expressed proteins (alpha = 0.04, fdr < 0.3) were inversely correlated (slope = − 0.77, *R*^2^ = 0.85), when comparing APPPS1 against spermidine (Fig. 3b). The opposite regulation highlights the beneficial effects of spermidine on AD-associated changes. No proteins were found to be regulated into the same direction, e.g., amplifying potentially disease-driving protein changes, indicating no adverse effects of spermidine on AD pathology (Fig. 3b, Additional file 1: Fig. S3a). To assess whether those proteins can discriminate the spermidine-treated groups from the controls, a principal component analysis (PCA) and hierarchical clustering was performed, resulting in a clear separation based on spermidine treatment (Additional file 1: Fig. S3b).

To consider also coordinated changes, which did not pass significance criteria on the single-protein level, we performed gene-set enrichment analysis (GSEA), a functional analysis with a special focus on biological process (GO:BP) and pathways (REACTOME) terms related to inflammation and neurodegeneration. In line with the transcriptomic results, spermidine treatment increased pathways involved in “microtubule cytoskeleton organization”, “regulation of actin filament binding” and “regulation of actin binding”, thus supporting the observed microglial migration changes upon spermidine treatment (Fig. 3c). While the transcriptomics analysis only revealed a few anti-inflammatory genes to be regulated by spermidine, a clear downregulation of many inflammatory pathways including “acute inflammatory response” as one of the top downregulated pathways, as well as IL-1 and IL-6 signaling and inflammasome pathways were found (Fig. 3c). Among the downregulated pathway clusters within a REACTOME enrichment map was a big cluster of IL signaling-related pathways indicating anti-inflammatory effects of spermidine. Matching previous studies [20, 46], spermidine also upregulated the pathway cluster “autophagy”. Furthermore, spermidine affected ubiquitin-associated pathways and SUMOylation (Fig. 3d). Comparing the pathways altered in APPPS1 mice with those found to be affected by spermidine revealed a clear reversal of the AD-associated induction of inflammation and oxidative phosphorylation and the downregulation of cytoskeletal pathways (Fig. 3e). Thus, we conclude that spermidine reverted AD-mediated effects in APPPS1 mice.

To assess whether these microglia-specific anti-inflammatory effects interfered with neuroinflammation later in the pathology, ten cytokines were quantified by electrochemiluminescence in brain homogenates of 290-day-old male spermidine-treated APPPS1 mice. Spermidine supplementation significantly reduced the AD-relevant pro-inflammatory cytokines IL-6, TNF-α, IL-12, IL-4 and IL-5 in 290-day-old mice (Fig. 3f), while not altering IL-1β (combined measurement of Pro-IL-1β and IL-1β), IFN-γ, IL-2, IL-10 and KC/GRO (Additional file 1: Fig. S3c), revealing indeed anti-inflammatory effects of spermidine in the CNS at late stages of disease pathology.

### Spermidine exhibits direct anti-inflammatory effects on microglia

**Fig. 4:**
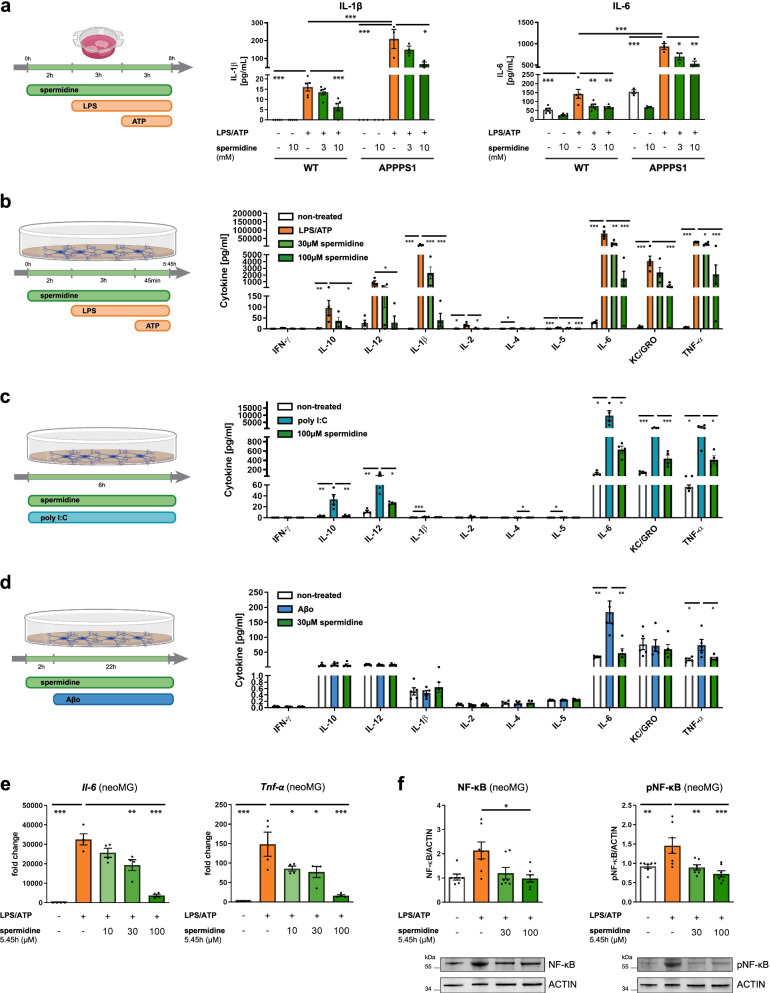
Spermidine exhibits direct anti-inflammatory effects on microglia. **a** Hemispheres of wild type (WT) and APPPS1 mice were coronally sliced and treated with the indicated spermidine concentration, LPS (10 µg/ml) and ATP (5 mM) as depicted. The IL-1β and IL-6 concentration in the supernatant was determined by ELISA; *n* = 3–5, two-way ANOVA, Tukey’s post hoc test. **b–f** Neonatal microglia (neoMG) were either treated with LPS (1 µg/ml) and ATP (2 mM), with poly I:C (50 µg/ml) or with oligomeric Aβ (Aβo, 5 µM) and the indicated spermidine concentrations as depicted in the schemes. **b–d** Amount of cytokines in the cell supernatant was determined by electrochemiluminescence (MesoScale Discovery panel); *n* = 4–5. **b** IFN-γ, IL-10, IL-12, IL-2: Kruskal–Wallis, Dunn’s multiple comparison; IL-1β, IL-4, IL-5, IL-6, KC/GRO, TNF-α: one-way ANOVA, Dunnett’s post hoc test. **c** INF-γ, IL-2, IL-4: Kruskal–Wallis, Dunn’s multiple comparison; IL-10, IL-12, IL-1β, IL-5, IL-6, KC/GRO, TNF-α: one-way ANOVA, Dunnett´s post hoc test. **d** IL-10, IL-12, IL-4, KC/GRO: Kruskal–Wallis, Dunn’s multiple comparison; INF-γ, IL-1β, IL-2, IL-5, IL-6, TNF-α: one-way ANOVA, Dunnett’s post hoc test. **e** The gene expression of *Tnf-α* and *Il-6* was assessed by RT-qPCR after treatment of neonatal microglia as depicted in **b**. Their expression was normalized to *Actin* and displayed as fold change compared to non-treated control cells; *n* = 4. *Il-6*: one-way ANOVA, Dunnett’s post hoc test; *Tnf-α:* Kruskal–Wallis, Dunn’s multiple comparison. **f** Levels of phosphorylated NF-κB (pNF-κB) and NF-κB were determined by western blot in neonatal microglia treated as depicted in **b**. Representative images are shown and protein levels are displayed as fold changes compared to non-treated controls normalized to ACTIN; *n* = 7. NF-κB: Kruskal–Wallis, Dunn’s multiple comparison; pNF-κB: one-way ANOVA, Dunnett’s post hoc test. *p < 0.05, **p < 0.01, ***p < 0.001

To assess whether spermidine influenced neuroinflammation in a direct manner or whether its effects on neuroinflammation were secondary, acute whole hemisphere slice cultures derived from 200-day-old WT or APPPS1 mice were treated with spermidine and subsequently stimulated with LPS and ATP (Fig. 4a). LPS/ATP treatment of APPPS1 slice cultures resulted in a massive release of IL-1β and IL-6 compared to slices from WT mice. Spermidine significantly reduced their levels in both genotypes (Fig. 4a), underlining that spermidine could directly influence neuroinflammation in APPPS1 mice.

To pinpoint which cytokine-producing glial cell accounts for the anti-inflammatory effects of spermidine, both neonatal microglia and astrocytes were activated either with LPS followed by ATP (Fig. 4b), with poly I:C (Fig. 4c) or oligomeric Aβ (Fig. 4d). The presence of spermidine reduced the LPS/ATP-induced release of IL-6, TNF-α, IL-5, IL-1β, IL-18, IL-10, IL-12, KC/GRO and IL-2 into the cell supernatant of microglia dose-dependently (Fig. 4b, Additional file 1: Fig. S4a). In addition, spermidine treatment reduced the poly I:C-induced release of IL-6, TNF-α, IL-10, IL-12, IL-4 and KC/GRO in neonatal microglia and astrocytes (Fig. 4c, Additional file 1: Fig. S4b, c). These anti-inflammatory effects were also confirmed in adult microglia (Additional file 1: Fig. S4d–h). Furthermore, spermidine reduced the IL-6 and TNF-α release by neonatal microglia upon treatment with oligomeric Aβ (Fig. 4d), also indicating a direct effect of spermidine on Aβ-induced neuroinflammation.

Mechanistically, spermidine reduced the gene expression of the Aβ-induced or LPS/ATP-induced AD-relevant cytokines *Il-6*, *Tnf-α* and *Il-1β* in neonatal microglia dose-dependently (Fig. 4e, Additional file 1: Fig. S4i, j). Also, poly I:C-induced gene expression of *II-6* and *Tnf-α* was decreased by spermidine in both neonatal microglia and astrocytes (Additional file 1: Fig. S4k, l). Reduced phosphorylation of NF-κB upon spermidine treatment might account for these gene expression changes (Fig. 4f, Additional file 1: Fig. S4m).

Correlating with the induction of autophagy as shown in the proteomic analysis of spermidine-treated APPPS1 mice, the anti-inflammatory effects of spermidine in vitro were autophagy-mediated. Spermidine induced expression of autophagic proteins significantly (Additional file 1: Fig. S5a–d) and no effects of spermidine treatment were measured upon autophagy activation with HBSS (Additional file 1: Fig. S5e–f). Impairment of autophagy by 3-MA or using primary microglia with BECN1 knockout (Tamoxifen-treated BECN1^flox/flox^·CX3CR1^CreERT2^ cultures) on the other hand, abolished spermidine-mediated effects (Additional file 1: Fig. S5g–i). Therefore, we concluded that spermidine exerts direct anti-inflammatory effects on microglia and astrocytes in an autophagy-dependent manner, correlating with the observed reduced neuroinflammation in 290-day-old APPPS1 mice.

### Spermidine regulates neuroinflammation beyond transcription by interfering with inflammasome assembly

**Fig. 5:**
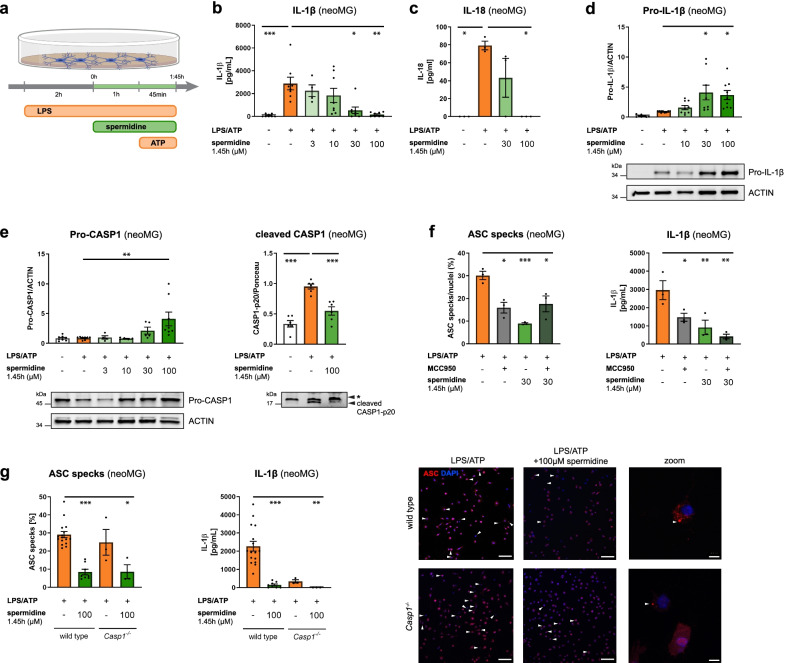
Spermidine regulates neuroinflammation beyond transcription by interfering with inflammasome assembly. Neonatal microglia (neoMG) were treated with LPS (1 µg/ml) and spermidine at indicated concentrations for 1.45 h and ATP (2 mM) as depicted in the scheme (**a**). **b** IL-1β concentration in the cell supernatant was determined by ELISA; *n* = 4–8; Kruskal–Wallis, Dunn´s multiple comparison. **c** IL-18 concentration in the cell supernatant was determined by ELISA; *n* = 3; Kruskal–Wallis, Dunn’s multiple comparison. **d** Pro-IL-1β protein levels were determined by western blot and normalized to ACTIN. Representative images are shown and values are displayed as fold changes compared to LPS/ATP-treated cells; *n* = 8–9; Kruskal–Wallis, Dunn’s multiple comparison. **e** Cellular Pro-CASP1 and cleaved CASP1 p20 levels in the supernatant were determined by western blot (* non-specific band). Pro-CASP1 was normalized to ACTIN (*n* = 4–8) and CASP1 p20 was normalized on whole protein content determined by Ponceau S staining (*n* = 3). Values are displayed as fold changes compared to LPS/ATP-treated cells; Pro-CASP1: Kruskal–Wallis, Dunn´s multiple comparison; cleaved CASP1: one-way ANOVA, Dunnett’s post hoc test. **f** Neonatal microglia were stimulated as shown in **a** and MCC950 was added 15 min before addition of ATP. Cells were stained for ASC to visualize inflammasomes and with DAPI for nuclear staining. The percentage of ASC specks in respect to the number of total cells (DAPI positive cells) was determined (left). The IL-1β concentration in the cell supernatant was assessed by ELISA (right); *n* = 3; one-way ANOVA, Dunnett’s post hoc test. **g** Neonatal WT and *Casp1*^−/−^ microglia were stimulated as shown in **a** but with 4 mM ATP to increase the number of inflammasomes. Cells were stained for ASC (red) to visualize inflammasomes and with DAPI (blue) for nuclear staining as shown in the representative images (scale bar = 100 µm). Arrowheads highlight ASC specks within microglia. The percentage of ASC specks in respect to the number of total cells (DAPI positive cells) was determined (left). The IL-1β concentration in the cell supernatant was assessed by ELISA (right); WT: *n* = 8–16; *Casp1*^−/−^: *n* = 3. Kruskal–Wallis, Dunn’s multiple comparison. *p < 0.05, **p < 0.01, ***p < 0.001

As recent studies and our proteome analysis showed that spermidine mediates some of its effects solely on protein level [20, 22, 31], the effects of spermidine on neuroinflammation beyond transcriptional control were studied by treating microglia with spermidine after activating/priming them with LPS (Fig. 5a). Interestingly, post-LPS spermidine treatment (1.45 h) reduced only the release of IL-1β and IL-18 into the cell supernatant (Fig. 5b, c) while not altering the release of all other measured cytokines (Additional file 1: Fig. S6a–c). *Il-1β*, *Il-6* and *Tnf-α* gene expression revealed no alterations by this spermidine treatment scheme (Additional file 1: Fig. S6d). However, increased protein levels of Pro-IL-1β and uncleaved Pro-CASP1 were found (Fig. 5d, e), indicating reduced processing at the NLRP3 inflammasome. This correlated with a reduction of cleaved and activated CASP1 and Gasdermin D (GSDMD), another substrate of CASP1, in the supernatant (Fig. 5e, Additional file 1: Fig. S6e).

While NLRP3 expression was not altered on the mRNA or protein level by spermidine (Additional file 1: Fig. S6f), staining and quantification of ASC specks/inflammasomes revealed that spermidine treatment reduced the number of ASC specks significantly (Fig. 5f, g). A similar reduction was also detected in *Casp1*^−/−^ microglia (Fig. 5g), indicating that spermidine did not directly interfere with Pro-CASP1 cleavage but rather with inflammasome formation. To test this hypothesis, the ASC-oligomerization inhibitor MCC950 [47] was added prior to adding ATP. No additive effects of MCC950 to the spermidine-mediated effects could be detected, underlining that spermidine was indeed interfering with ASC-oligomerization and inflammasome formation (Fig. 5f). Consistent with this hypothesis, western blot analysis for ASC after chemical crosslinking showed reduced appearance of ASC oligomers in spermidine-treated cells (Additional file 1: Fig. S6g), while the amount of ASC monomers was not altered (Additional file 1: Fig. S6h). Thus, spermidine treatment of activated microglia reduced IL-1β processing by interfering with the oligomerization of ASC-positive inflammasomes.

Taken together, we elucidated a novel regulatory mechanism of spermidine in addition to targeting NF-κB-mediated transcription of pro-inflammatory genes. Thus, spermidine targets multifarious pathways, such as degradation of Aβ, proliferation and active reduction of inflammatory signaling, which stabilizes a presumably protective microglia population.

## Discussion

Delaying AD progression still presents an urgent unmet clinical need. Based on recent advances in our understanding of AD pathogenesis that resulted in the appreciation of the impact of neuroinflammation and autophagy, we assessed the therapeutic effects of the autophagic activator spermidine on Aβ pathology in APPPS1 mice.

Interestingly, spermidine treatment significantly increased Aβ degradation and reduced soluble Aβ levels in vivo, while Aβ plaque burden and size were not altered. Whereas the effects of spermidine on AD pathology have not been assessed so far, De Risi et al. [48] reported that spermidine decreased soluble Aβ and α-synuclein in a mouse model with mild cognitive impairment. Despite the fact that the toxicity and importance of soluble vs. insoluble Aβ in AD pathology is still a matter of debate, there is clear evidence that soluble Aβ causes more synaptotoxicity than plaque-bound insoluble Aβ. It was shown to alter synaptic transmission and to mediate synaptic loss and neuronal death [49–51], thus suggesting that targeting soluble Aβ might suffice to ameliorate AD. In line with this, recent data suggest that microglia create core plaques as a protective measure to shield the brain from soluble Aβ. The DAM marker AXL is thought to contribute to this formation [52]. In addition, the AXL-GAS6 pathway has been shown to not only suppress the microglial inflammatory response but also to mediate Aβ phagocytosis [44]. Thus, the increased AXL levels in spermidine-treated APPPS1 mice as well as the in vitro phagocytosis experiments underline that spermidine affects microglial phagocytosis and degradation of Aβ. As the APPPS1 mouse model exhibits a fast disease progression with a strong genetically driven Aβ pathology appearing at 60 days, the substantial effect of spermidine on soluble Aβ highlights its potential to interfere with AD progression. Since neuroinflammation is a known driver for plaque formation [5, 6], the additional anti-inflammatory effects of spermidine may have a beneficial effect on insoluble Aβ and plaques in mice older than those we analyzed within the frame of this work (namely, older than 290 days).

Notably, previous studies assessing the effect of autophagy activation on AD pathology also revealed reduced Aβ pathology [53–56]; however, the mechanisms by which autophagy modulation targets AD pathology had not been elucidated so far. By applying snRNA-seq to 180-day-old spermidine-treated APPPS1 mice, we revealed microglia as the main glial cell type to be targeted by spermidine. The most profound effects of spermidine on the transcriptome level were seen in the AD-associated microglia cluster 2, which was characterized by increased migration, cell motility, phagocytosis and cell proliferation. While acute activation of microglia in early disease pathology induces microglial phagocytosis and migration towards plaques, later stages of AD pathology and chronic priming of microglia with Aβ have adverse effects [57, 58]. In accordance, microglial motility in the presence of Aβ plaques was found to be decreased in APPPS1 mice compared to control mice when a focal laser lesion was induced [59].

A common denominator of the transcriptome and proteome analysis was that spermidine prevented AD-associated cytoskeletal changes and thus, might increase microglial migration and cell motility, as demonstrated in vitro. Accordingly, spermidine was found to promote cell migration in neural cells and keratinocytes as well as wound healing processes ex vivo and in vivo [60]. In line with previous publications [61], the proteomics analysis revealed that spermidine also preserved the energy metabolism in microglia from APPPS1 mice by affecting oxidative phosphorylation, glycolysis and gluconeogenesis. By promoting genes and proteins involved in cell motility, migration and phagocytosis, spermidine seems to delay the onset of the late-stage AD-associated microglial population.

Interestingly, some of those changes might be exerted by SUMOylation. SUMOylation is, similar to ubiquitination, a post-translational modification regulating transcription, cell proliferation and protein stability and turnover. To our knowledge this is the first time that an effect of spermidine on SUMOylation is described and it may also contribute to autophagy induction and/or protein degradation. In correlation, recent publications on the post-translational modification called hypusination [31, 32] indicate that spermidine can exert some of its function on the post-transcriptional levels.

In addition, spermidine also increased the abundance of microglia cluster 2. Although it is still under discussion whether proliferation of microglia in AD is beneficial or detrimental [62], spermidine mediated the enlargement of a microglial subpopulation showing increased phagocytosis and cell motility including *Axl* expression as described above. Several regulated genes, such as *Arpc3* [63], *Glp2r* [64], *Sirt3* [65] and *Per1* [66, 67] have been reported to exert protective effects in neurodegenerative diseases or reverse memory deficits in various models, underlining the observed protective effects of spermidine. For instance, SIRT3 was shown to target similar pathways as spermidine, such as inflammation, including the IL-1β processing pathway [42, 43] and microglial migration [68]. Even though microglia were the main glial cells to be affected by spermidine on transcriptional level at 180 days, our in vitro analyses revealed that spermidine also reduced cytokine production in astrocytes, indicating that astrocytes might also be altered at later stages of AD pathology. While only few anti-inflammatory effects of spermidine were found by snRNA-seq, the proteomics analysis of microglia isolated from spermidine-treated APPPS1 revealed a clear downregulation of inflammatory pathways. These changes might pave the path for the brain-wide reduction in cytokines mediated by spermidine at 290 days, when APPPS1 mice are known to exhibit profound neuroinflammation. Of note, while spermidine was found to target the IL-1β processing pathway at 180 days and in vitro, no changes in IL-1β cytokine levels were found in 290-day-old APPPS1 mice. This might be due to the fact that the MSD cytokine panel does not distinguish between Pro-IL-1β and cleaved IL-1β. Next to the in vitro effects of spermidine on transcription of cytokines by modulating the NF-κB signaling pathway, which was previously described in BV2 and macrophages [29, 30], we identified a novel spermidine-modulated post-translational mechanism. Spermidine interfered with the ASC assembly of the NLRP3 inflammasome and thereby reduced the production of IL-1β. This pathway was also found to be downregulated in the proteomics analysis of spermidine-treated APPPS1 microglia, again indicating that spermidine acts beyond modulation of transcription.

## Conclusions

Activators of autophagy such as fasting or caloric restriction, exercise, rapamycin, an inhibitor of the mechanistic target of rapamycin (mTOR), and metformin were shown to prolong the life span of several species and to reduce Aβ deposition in different mouse models [53–56], yet most of these drugs—in contrast to the orally applicable spermidine—were problematic in terms of tolerability and/or administration. Therefore, the body-endogenous substance spermidine seems to be an attractive therapeutic dietary supplement as it attenuated AD-relevant neuroinflammation, reduced synaptotoxic soluble Aβ and reverted AD-associated proteomic changes with no adverse effects. Since spermidine supplementation is already tested in humans, the extension of spermidine supplementation from individuals with subjective cognitive decline [32, 69–71] to clinical trials aimed at testing spermidine efficacy in AD patients appears to be a tempting approach.

## Supplementary Information

**Additional file 1.** Supplementary figures.

**Additional file 2.** Supplementary methods.

**Additional file 3.** Supplementary Table 1.

## Data Availability

Data generated and analyzed during this study are included in this published article and its additional information files. The snRNA-seq dataset has been deposited in the GEO database with the accession identifier GSE206202: https://www.ncbi.nlm.nih.gov/geo/query/acc.cgi?acc=GSE206202. The mass spectrometry proteomics dataset has been deposited with the ProteomeXchange Consortium via the PRIDE partner repository with the dataset identifier PXD034638.

## Abbreviations

AD: Alzheimer’s disease
Aβ: Amyloid β
APP: Amyloid precursor protein
DAM: Disease-associated microglia
IL: Interleukin
LPS: Lipopolysaccharide
neoMG: Neonatal microglia
AdMG: Adult microglia
neoAC: Neonatal astrocytes
snRNA-seq: Single nuclei sequencing
WT: Wild type
