# Supplementary material for: Spermidine reduces neuroinflammation and soluble amyloid beta in an Alzheimer’s disease mouse model
Source: J Neuroinflammation. 2022 Jul 2;19:172. doi: 10.1186/s12974-022-02534-7 (PMC9250727; doi:10.1186/s12974-022-02534-7)
Supplement: Supplementary file 1 — Additional file 1. Supplementary figures. [file 12974_2022_2534_MOESM1_ESM.pdf]

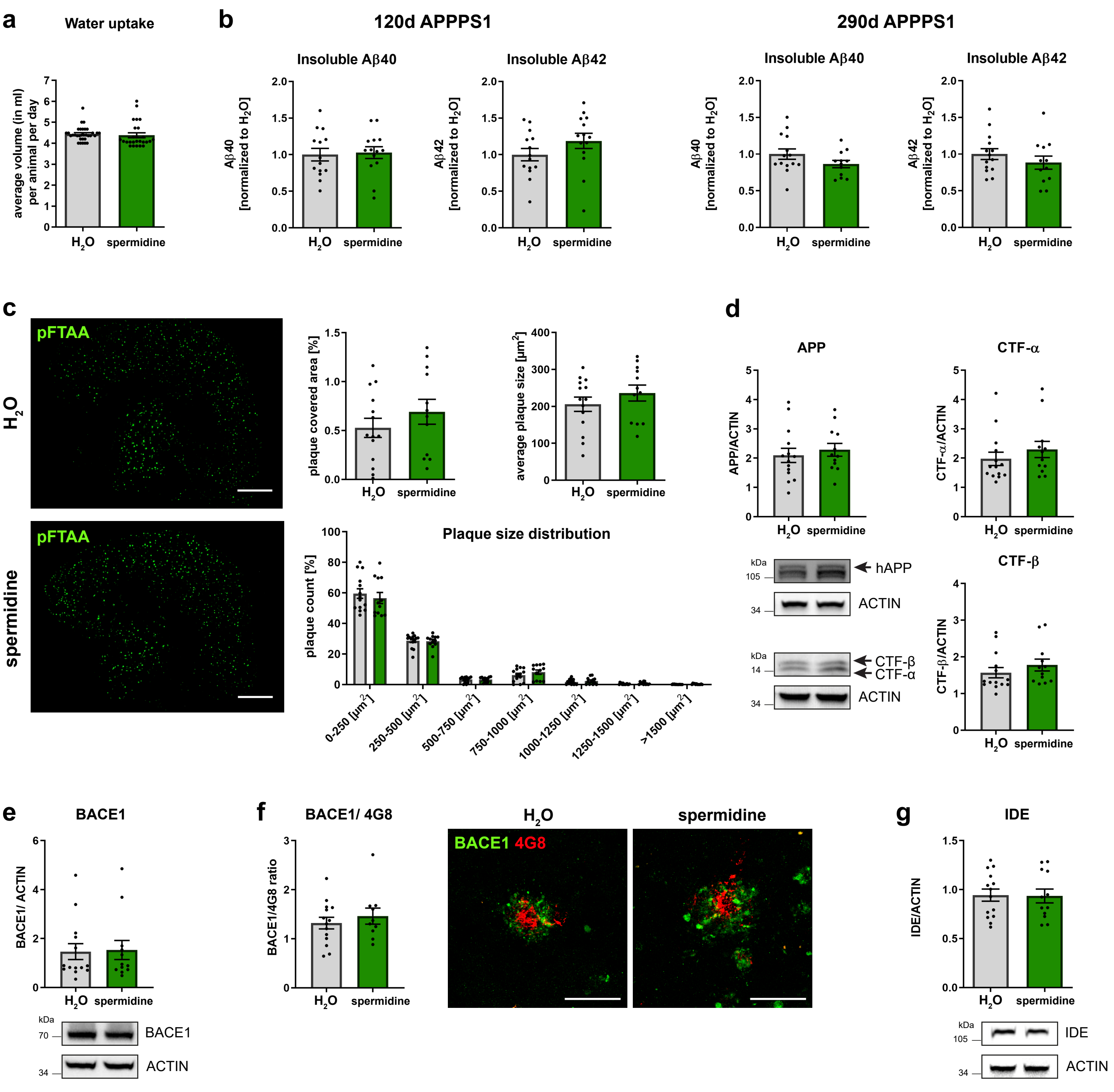

Fig. S1

APPPS1 mice were treated with 3 mM spermidine via their drinking water as depicted in Fig. 1a and analyzed at an age of 120 or 290 days (mixed sex). Spermidine-treated APPPS1 mice were compared to non-treated controls (H_2O). **a** The average volume in ml per animal per day was calculated based on the volume that was drank in an interval of 3.5 days per cage. APPPS1 H_2O (n = 32), APPPS1 spermidine (n = 26); Mann-Whitney-U test. **b** The Aβ40 and Aβ42 content was measured in the SDS (insoluble) fraction of brain homogenates of 120- or 290-day-old spermidine-treated mice and water controls using electrochemiluminescence (MesoScale Discovery panel). Values were normalized to water controls. 120d APPPS1 H_2O (n = 14), 120d APPPS1 spermidine (n = 14), 290d APPPS1 H_2O (n = 14), 290d APPPS1 spermidine (n = 12); two-tailed t-test. **c** Aβ plaques in tissue sections of 290-day-old mice were stained with pFTAA and the plaque covered area (%), the average plaque size and a plaque size distribution of the cortex was determined by ImageJ analysis; two-tailed t-test. The number of plaques of each indicated size is displayed as a percentage of the total number of plaques detected; Mann-Whitney-U test. APPPS1 H_2O (n = 14), APPPS1 spermidine (n = 12). Scale bar = 1 mm. **d** The levels of APP, CTF-α and CTF-β were determined by western blot of the TX (membrane-bound) protein fraction of 290-day-old spermidine-treated APPPS1 mice. Representative western blot images are shown. APPPS1 H_2O (n = 14), APPPS1 spermidine (n = 12); Mann-Whitney-U test. **e** The levels of BACE1 were determined by western blot of the TX (membrane-bound) protein fraction of 290-day-old spermidine-treated APPPS1 mice. Representative western blot images are shown. APPPS1 H_2O (n = 14), APPPS1 spermidine (n = 12); Mann-Whitney-U test. **f** Tissue sections of 290-day-old mice were stained for BACE1 (green) and 4G8-positive plaques (red). The BACE1-covered area normalized to 4G8-positive area was determined. Scale bar = 50 µm. APPPS1 H_2O (n = 14), APPPS1 spermidine (n = 12); two-tailed t-test. **g** The levels of IDE were determined by western blot of the TX (membrane-bound) protein fraction of 290-day-old spermidine-treated APPPS1 mice. Representative western blot images are shown. APPPS1 H_2O (n = 14), APPPS1 spermidine (n = 12); two-tailed t-test.

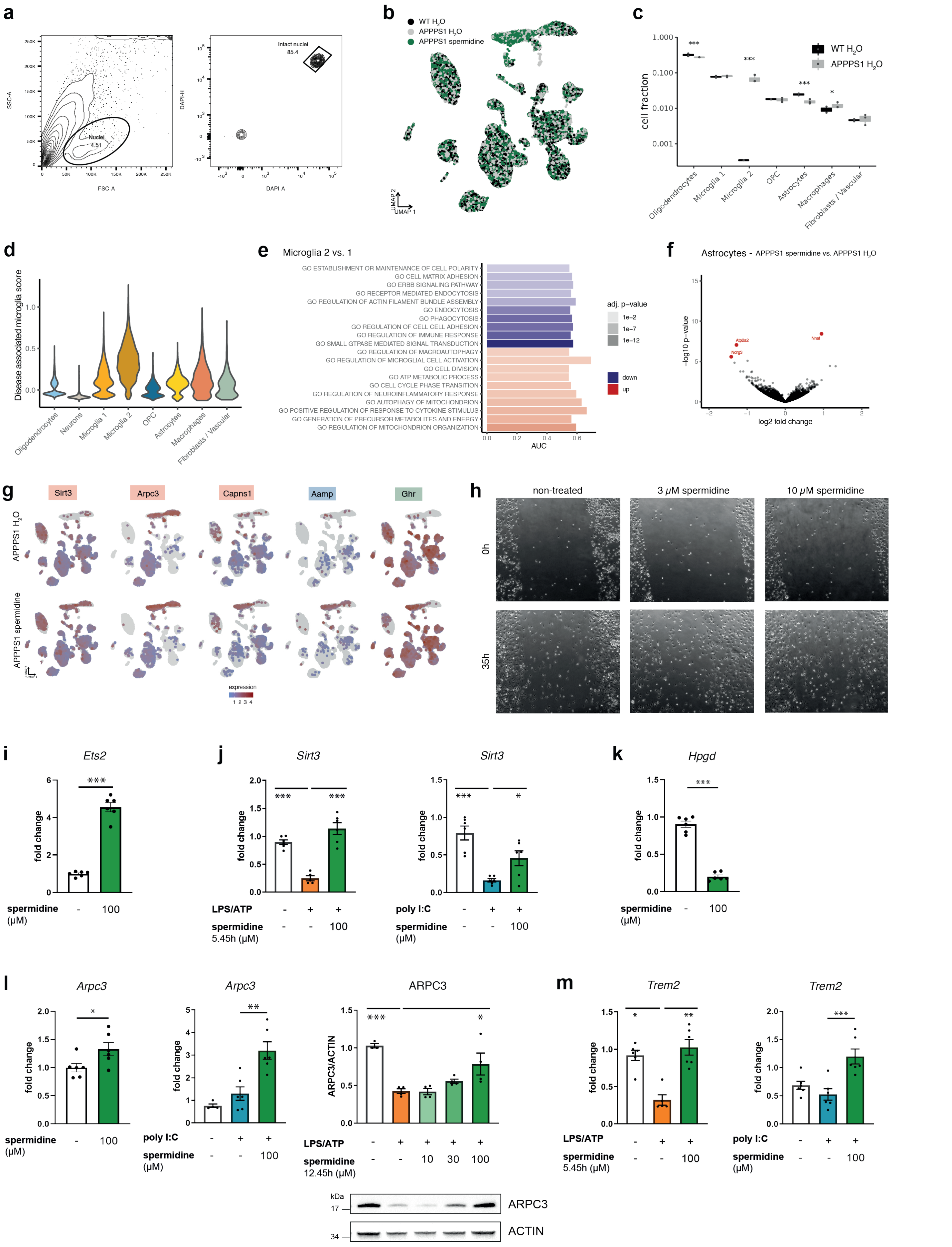

Fig. S2

Male APPPS1 mice were treated with 3 mM spermidine via their drinking water and analyzed at an age of 180 days. **a** Whole hemisphere homogenates were stained with DAPI and intact DAPI-stained nuclei were sorted using a FSC/SSC based gate to exclude debris followed by exclusion of damaged nuclei in a DAPI-A/DAPI-H for subsequent single nuclei sequencing. **b** UMAP embedding of snRNAseq data with genotypes indicated. **c** Cluster abundance in APPPS1 H_2O and WT H_2O mice. P-values from mixed-effects binomial model. **d** Violin plot of a gene signature score for disease-associated microglia (using *Apoe, Axl, B2m, Ccl6, Ccl6, Ccl6, Cd9, Clec7a, Csf1, Cst7, Ctsb, Ctsd, Fth1, Itgax, Lyz2, Timp2, Trem2* and *Tyrobp*). **e** Selected terms from a gene set enrichment analysis of differential expression between microglia clusters 1 and 2 using tmod. **f** Volcano plot of genes differentially expressed in astrocytes of spermidine-treated APPPS1 versus H_2O APPPS1 mice. Genes with adj. p-value < 0.01 are highlighted in red and top 5 up- and down-regulated genes are indicated. **g** Expression of *Sirt3, Arpc3, Capns1, Aamp* and *Ghr* in spermidine-treated APPPS1 (bottom) and H_2O APPPS1 mice (top). Color scale indicates normalized expression, grey dots represent no data. **h** Representative images of a wound healing/-scratch assay performed with spermidine-treated neonatal microglia at time point 0 h and 35 h. Quantification is shown in Fig. 1h. **i** Neonatal microglia were treated with 100 µM spermidine for 6 h and the gene expression of *Ets2* was assessed by RT-qPCR. Its expression was normalized to *Actin* and displayed as fold change compared to non-treated control cells; n = 6, two-tailed t-test. **j** Neonatal microglia were treated with the indicated concentrations of spermidine in combination with LPS (1 µg/ml) and ATP (2 mM) or with poly I:C (50 µg/ml) and *Sirt3* was assessed by RT-qPCR (right panels), normalized to *Actin* and displayed as fold change compared to non-treated control cells; n = 5-6, one-way ANOVA, Dunnett´s post hoc test. **k** Neonatal microglia were treated with 100 µM spermidine for 6 h and the gene expression of *Hpgd* was assessed by RT-qPCR. Its expression was normalized to *Actin* and displayed as fold change compared to non-treated control cells; n = 6, two-tailed t-test. **l** Neonatal microglia were treated with the indicated amount of spermidine in combination with LPS (1 µg/ml) and ATP (2 mM) for 5.45 h for qPCR analysis and for 12.45 h for western blot analysis. The gene expression was assessed by RT-qPCR (left panels) and the *Arpc3* expression was normalized to *Actin* and displayed as fold change compared to non-treated control cells; n = 5-6. ARPC3 protein levels were determined by western blot and normalized to ACTIN. Representative images are shown and values are displayed as fold changes compared to non-treated cells; n = 4, one-way ANOVA, Dunnett´s post hoc test. **m** Neonatal microglia were treated with the indicated concentrations of spermidine in combination with LPS (1 µg/ml) and ATP (2 mM) or with poly I:C (50 µg/ml) and the gene expression of *Trem2* was assessed by RT-qPCR. Its expression was normalized to *Actin* and displayed as fold change compared to non-treated control cells; n = 5-6. LPS/ATP: Kruskal-Wallis, Dunn´s multiple comparison; poly I:C: one-way ANOVA, Dunnett´s post hoc test. * P < 0.05, ** P < 0.01, *** P < 0.01.

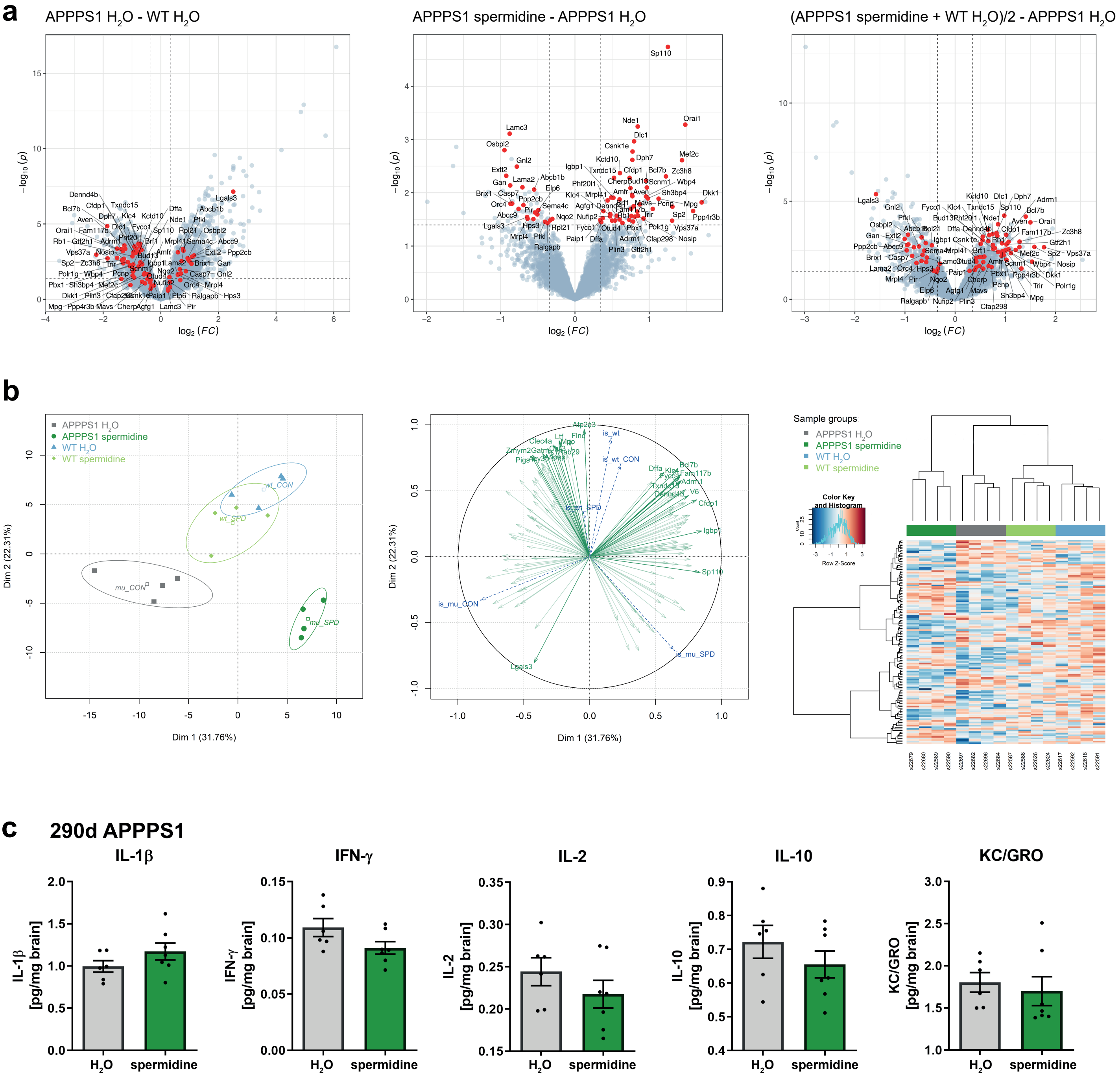

Fig. S3

a Volcano plots for three contrasts: contrast 1 = APPPS1 H_2O – WT H_2O (AD model effect, left panel), contrast 2 = APPPS1 spermidine – APPPS1 H_2O (spermidine effect, central panel), and contrast 5 = (contrast 2 – contrast 1)/2 (spermidine anti-AD effect, right panel). On all three plots proteins that are simultaneously significantly regulated (alpha = 0.04) in two contrasts, contrast 2 (spermidine effect) and contrast 5 (spermidine anti-AD effect), are labelled. Dashed horizontal line shows the significance level use for feature selection. Vertical dashed lines show logFC threshold = median SD of linear modelling. **b** Post hoc PCA analysis and hierarchical clustering using features significantly regulated (alpha = 0.04 and FC threshold = 1.27) in contrast 2 (spermidine effect). Left panel represents PCA score plot demonstrating clear separation of APPPS1 spermidine samples from APPPS1 H_2O samples in the first two principal components (PCs). Middle panel represents the PCA loading plot and shows top 25 proteins correlation with the first two PCs. Right panel shows heatmap with results of hierarchical clustering of samples in the space of features regulated in contrast 2. **c** The content of the cytokines in the TBS (soluble) fraction of brain homogenates of male 290-day-old spermidine-treated APPPS1 mice was measured using electrochemiluminescence (MesoScale Discovery panel). APPPS1 H_2O ($n = 6$), APPPS1 spermidine ($n = 7$). IL-1β, IFN-γ, IL-2, IL-10: two-tailed t-test; KC/GRO: Mann-Whitney-U test.

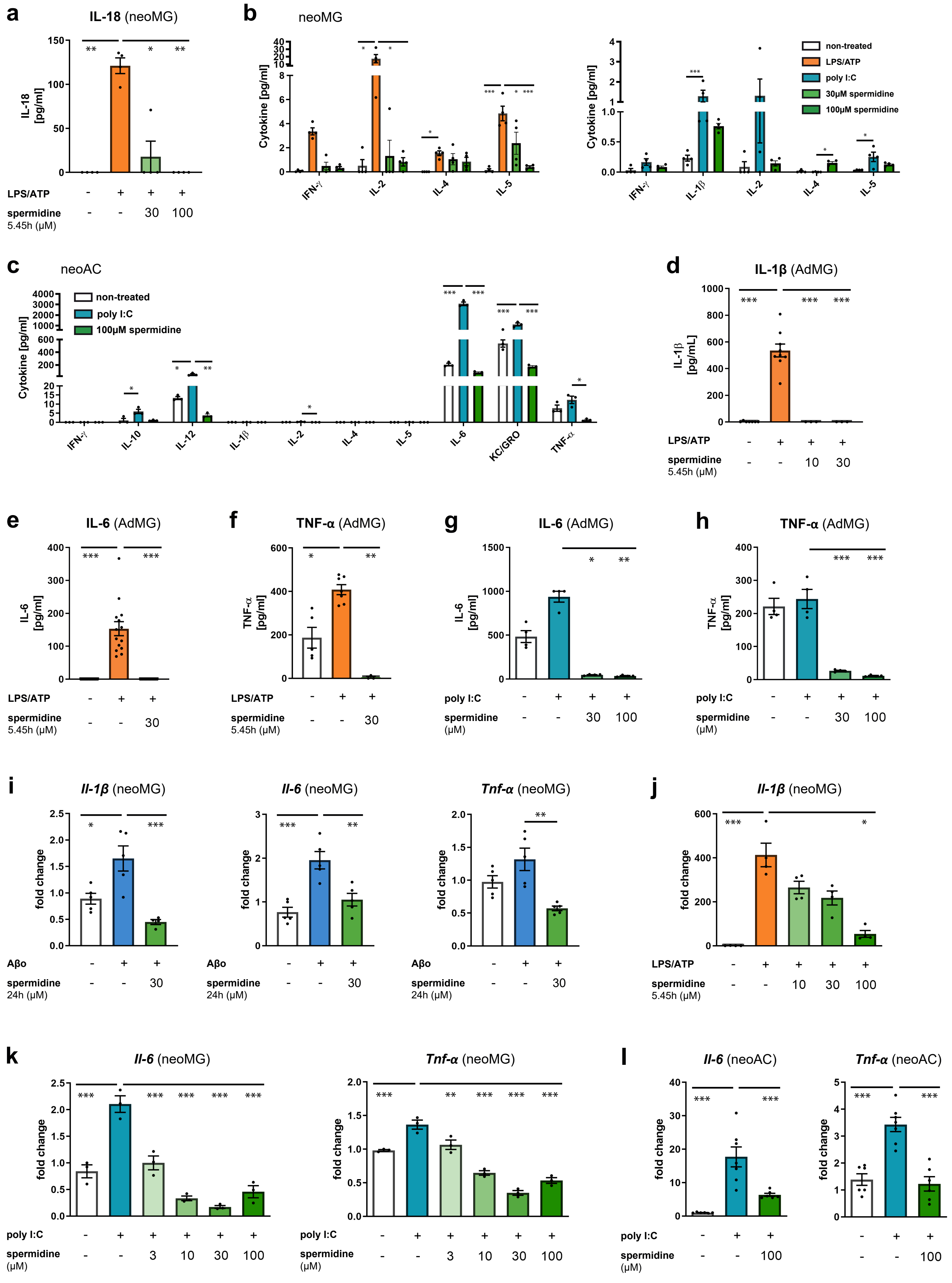

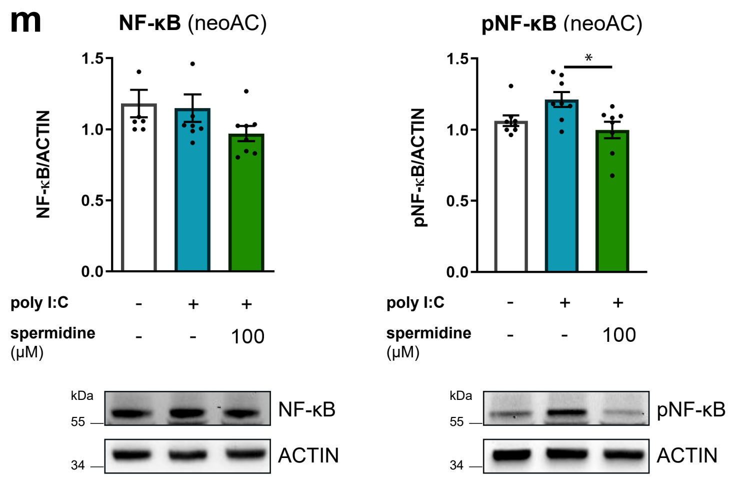

Fig. S4

Neonatal microglia (neoMG), adult microglia (AdMG) and neonatal astrocytes (neoAC) were either treated with LPS (1 µg/ml) and ATP (2 mM), with poly I:C (50 µg/ml) or with oligomeric Aβ (Aβo, 5 µM) and the indicated spermidine concentrations as depicted in Fig. 4b, Fig. 4c or Fig. 4d. **a** The concentration of IL-18 in the cell supernatant after LPS/ATP and spermidine treatment was measured by ELISA; n = 4; Kruskal-Wallis, Dunn´s multiple comparison. **b** The amount of cytokines in the cell supernatant was determined by electrochemiluminescence (MesoScale Discovery panel). Cytokines with low abundance displayed in Fig. 4b and Fig. 4c are shown; n = 4-5. LPS/ATP: IFN-γ, IL-2: Kruskal-Wallis, Dunn´s multiple comparison; IL-4, IL-5: One-way ANOVA, Dunnett´s post hoc test. poly I:C: INF-γ, IL-2, IL-4: Kruskal-Wallis, Dunn´s multiple comparison; IL-1β, IL-5: One-way ANOVA, Dunnett´s post hoc test. **c** The amount of cytokines in the cell supernatant of neonatal astrocytes treated with spermidine and poly I:C was determined by electrochemiluminescence (MesoScale Discovery panel); n = 3. IFN-γ, IL-12, IL-1β, IL-2, IL-4, IL-5, IL-6, KC/GRO: one-way ANOVA, Dunnett´s post hoc test; IL-10, TNF-α: Kruskal-Wallis, Dunn´s multiple comparison. **d-h** Adult microglia (AdMG) were isolated with magnetic activated cell sorting from 160-day-old WT mice. **d-f** The concentration of IL-1β, IL-6 and TNF-α in the cell supernatant treatment after LPS/ATP and spermidine treatment was measured by ELISA; n = 3-9; Kruskal-Wallis, Dunn´s multiple comparison. **g-h** The concentration of IL-6 and TNF-α in the cell supernatant after poly I:C and spermidine treatment was measured by ELISA; n = 4. TNF-α: one-way ANOVA, Dunnett´s post hoc test. IL-6: Kruskal-Wallis, Dunn´s multiple comparison. **i** The mRNA expression levels of *Il-1β, Il-6* and *Tnf-α* after oligomeric Aβ and spermidine treatment of neonatal microglia was measured by RT-qPCR; n = 5; one-way ANOVA, Dunnett´s post hoc test. **j** The mRNA expression levels of *Il-1β* after LPS/ATP and spermidine treatment of neonatal microglia was measured by RT-qPCR; n = 4; Kruskal-Wallis, Dunn´s multiple comparison. **k** The mRNA expression levels of *Il-6* and *Tnf-α* after poly I:C and spermidine treatment of neonatal microglia was measured by RT-qPCR; n = 3; one-way ANOVA, Dunnett´s post hoc test. **l** The mRNA expression levels of *Il-6* and *Tnf-α* after poly I:C and spermidine treatment of neonatal astrocytes was measured by RT-qPCR; n = 6-7; one-way ANOVA, Dunnett´s post hoc test. **m** Levels of phosphorylated NF-κB (pNF-κB) and NF-κB were determined by western blot in neonatal astrocytes treated with poly I:C and spermidine. Representative images are shown and protein levels are displayed as fold changes compared to non-treated controls normalized to ACTIN; n = 7; NF-κB: Kruskal-Wallis, Dunn´s multiple comparison; pNF-κB: one-way ANOVA, Dunnett´s post hoc test. * $P < 0.05$, ** $P < 0.01$, *** $P < 0.001$.

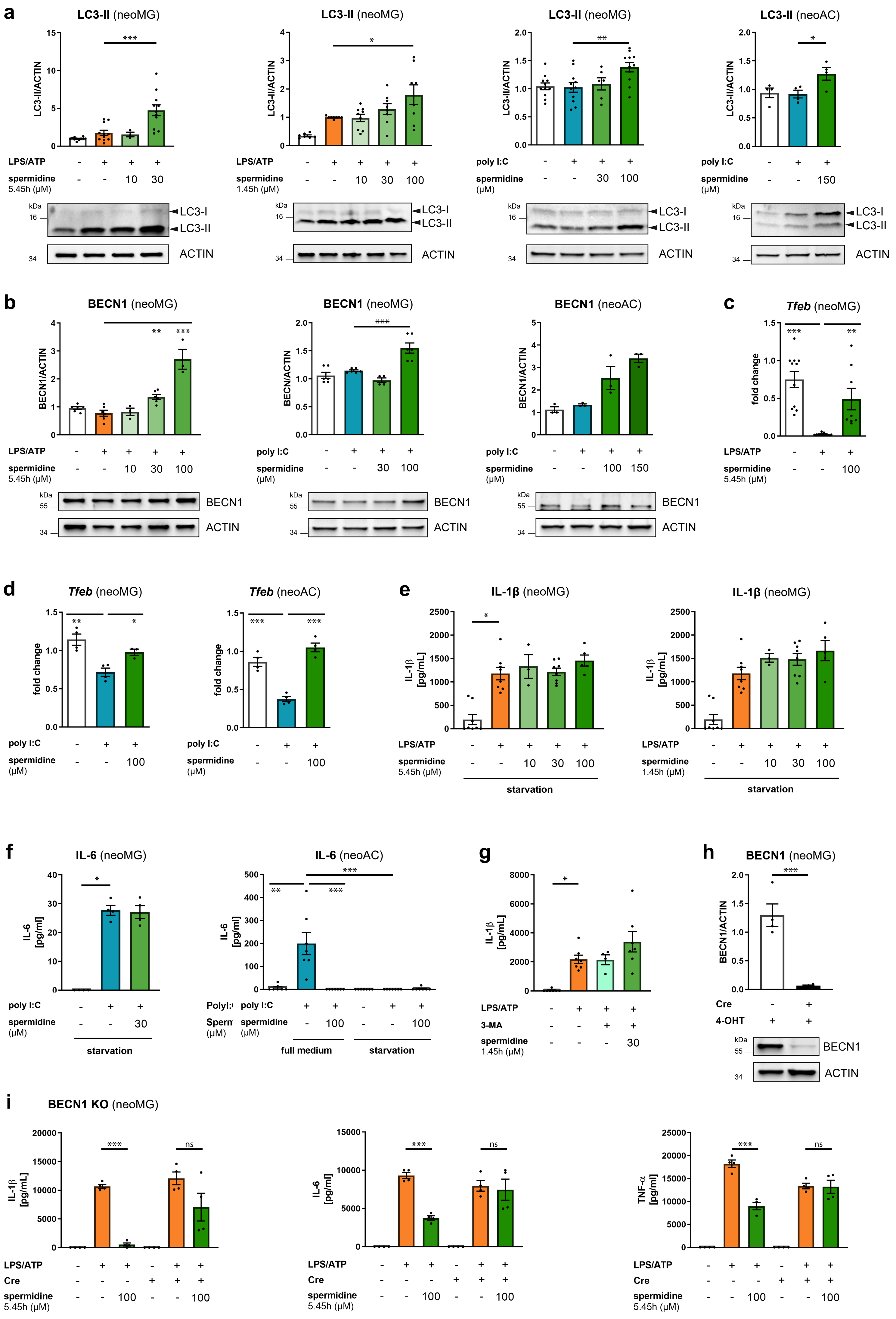

Fig. S5

Neonatal microglia (neoMG) and astrocytes (neoAC) were stimulated with LPS (1 µg/ml) and ATP (2 mM) or with poly I:C (50 µg/ml) and the indicated spermidine concentrations as depicted in Fig. 4b, 4c or 5a. **a** LC3-II levels were determined by western blot and normalized to ACTIN. Representative images are shown and values are displayed as fold changes compared to non-treated controls; microglia LPS/ATP 5.45h n = 3-11; microglia LPS/ATP 1.45h n = 8-9; microglia poly I:C n = 6-11; astrocytes poly I:C n = 4. One-way ANOVA, Dunnett´s post hoc test. **b** BECN1 levels were determined by western blot and normalized to ACTIN. Representative images are shown and values are displayed as fold changes compared to non-treated controls; microglia LPS/ATP n = 3-6; microglia poly I:C n = 6; astrocytes poly I:C n = 3. neoMG: one-way ANOVA, Dunnett´s post hoc test; neoAC: Kruskal-Wallis, Dunn´s multiple comparison. **c-d** The gene expression of *Tfeb* was assessed by RT-qPCR. Its expression was normalized to *Actin* and displayed as fold change compared to non-treated control cells; microglia LPS/ATP n = 8-11; microglia poly I:C n = 3-4; astrocytes poly I:C n = 4. LPS/ATP: Kruskal-Wallis, Dunn´s multiple comparison; poly I:C: one-way ANOVA, Dunnett´s post hoc test. **e-f** Cells were stimulated in starvation medium HBSS and the IL-1β or IL-6 concentration in the cell supernatant was determined by ELISA; microglia LPS/ATP 5.45h n = 3-8; microglia LPS/ATP 1.45h n = 3-8; microglia poly I:C n = 4; astrocytes poly I:C n = 7. Kruskal-Wallis, Dunn´s multiple comparison. **g** Neonatal microglia were stimulated as in Fig. 5a and 3-MA was added simultaneously with spermidine. The IL-1β concentration in the cell supernatant was determined by ELISA; n = 7; Kruskal-Wallis, Dunn´s multiple comparison. **h** Neonatal BECN1^{flox_flox}.CX3CR1^{CreERT2} microglia positive or negative for Cre were treated with Tamoxifen and BECN1 levels were assessed by western blot and normalized to ACTIN. Representative images are shown and values are displayed as fold changes compared to non-treated controls; n = 4; two-tailed t-test. **i** Neonatal BECN1^{flox_flox}.CX3CR1^{CreERT2} microglia positive or negative for Cre were treated with Tamoxifen (4-OHT) and subsequently treated as depicted in Fig. 4b. The release of IL-1β, IL-6 and TNF-α was determined by ELISA; n = 3; two-tailed t-test. * P < 0.05, ** P < 0.01, *** P < 0.001.

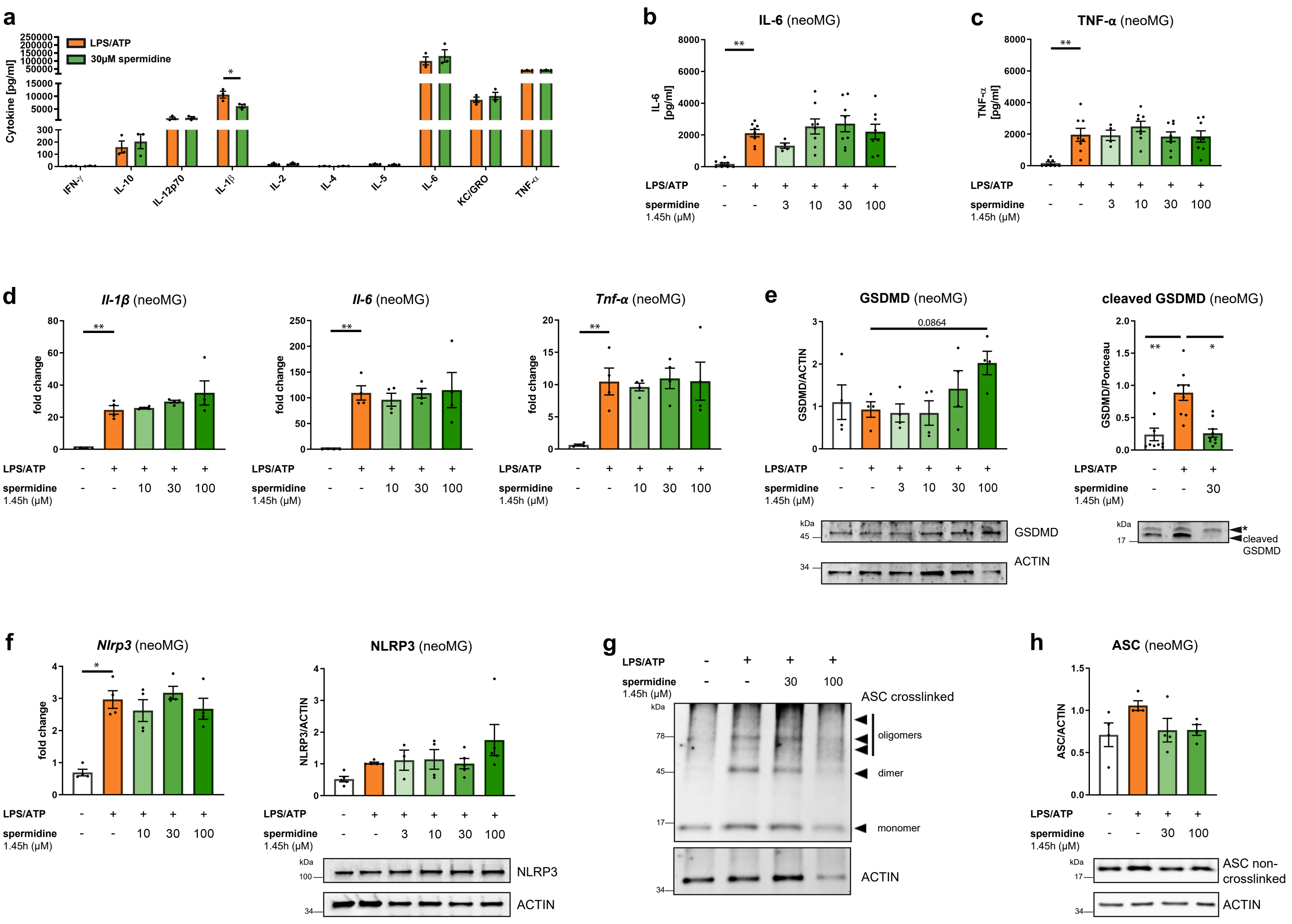

Fig. S6

Neonatal microglia (neoMG) were treated with LPS/ATP and spermidine as shown in Fig. 5a. **a** The amount of cytokines in the cell supernatant was determined by electrochemiluminescence (MesoScale Discovery panel); n = 3. IL-12, IL-1β, IL-2, IL-5, IL-6, KC/GRO, TNF-α: one-way ANOVA, Dunnett´s post hoc test; IFN-γ, IL-10, IL-4: Kruskal-Wallis, Dunn´s multiple comparison. **b** The IL-6 concentration in the cell supernatant was determined by ELISA; n = 4-8; Kruskal-Wallis, Dunn´s multiple comparison. **c** The TNF-α concentration in the cell supernatant was determined by ELISA; n = 4-8; Kruskal-Wallis, Dunn´s multiple comparison. **d** The gene expression of *Il-1β, Tnf-α* and *Il-6* was assessed by RT-qPCR. The expression was normalized to *Actin* and displayed as fold change compared to non-treated control cells; n = 4; one-way ANOVA, Dunnett´s post hoc test. **e** Cellular and cleaved GSDMD (C-terminal fragment) levels in the supernatant were determined by western blot (* non-specific band). GSDMD was normalized to ACTIN (n = 4) and the C-terminal fragment was normalized to whole protein content determined by Ponceau S staining (n = 9). Values are displayed as fold changes compared to LPS/ATP-treated cells; one-way ANOVA, Dunnett´s post hoc test for GSDMD and Kruskal-Wallis, Dunn´s multiple comparison for cleaved GSDMD. **f** NLRP3 protein expression was determined by western blot and normalized to ACTIN. Representative images are shown and values are displayed as fold changes compared to LPS/ATP-treated cells; n = 3-5. The gene expression of *Nlrp3* was assessed by RT-qPCR. Its expression was normalized to *Actin* and displayed as fold change compared to non-treated control cells; n = 4; Kruskal-Wallis, Dunn´s multiple comparison. **g** Proteins were chemically cross-linked by DSS. ASC monomer and oligomers were visualized by western blot.
h ASC monomer protein expression was determined by western blot and normalized to ACTIN. Representative images are shown and values are displayed as fold changes compared to LPS/ATP-treated cells; n = 4; Kruskal-Wallis, Dunn´s multiple comparison. * P < 0.05, ** P < 0.01, *** P < 0.001.
