# Supplementary material for: Spermidine reduces neuroinflammation and soluble amyloid beta in an Alzheimer’s disease mouse model
Source: J Neuroinflammation. 2022 Jul 2;19:172. doi: 10.1186/s12974-022-02534-7 (PMC9250727; doi:10.1186/s12974-022-02534-7)
Supplement: Supplementary file 2 — Additional file 2. Supplementary methods. [file 12974_2022_2534_MOESM2_ESM.docx]

Histology

Paraformaldehyde-fixed and sucrose-treated hemispheres were frozen and cryosectioned coronally at 40 µm using a cryostat (Thermo Scientific HM 560) and stored afterwards in cryoprotectant (0.65 g NaH2PO4 × H_2_O, 2.8 g Na2HPO4 in 250 ml ddH_2_O, pH 7.4 with 150 ml ethylene glycol, 125 ml glycerine) at 4°C until staining.

For immunohistochemistry, sections were washed with PBS and incubated with pentameric formyl thiophene acetic acid (pFTAA, 1:500, Sigma-Aldrich) for 30 min at RT. Subsequently, cell nuclei were counterstained with DAPI (1:2000, Roche, 10236276001) and sections embedded in fluorescent mounting medium (Dako, S3023). For quantification of pFTAA positive Aβ plaques, images of 10 serial coronal sections per animal were taken with an Olympus BX53 microscope, equipped with the QImaging camera COLOR 12 BIT and a stage controller MAC 6000 (1.25x objective). Images were analyzed using ImageJ by defining the cortex as the region of interest. Images were converted to grey scale and by using the same threshold for all sections, the pFTAA-positive area [in %] was obtained. Additionally, the average plaque size was determined and further analyzed by performing a plaque size distribution using thresholds for the size of the pFTAA-positive particles. The average of all 10 sections per animal was displayed in the graphs.

For fluorescent double staining for AXL and IBA1, sections were blocked for 1 h at RT in 1x PBS with 0.3% Triton-X 100, 0.1% Tween-20, 5% donkey serum and 2% BSA and incubated with the primary antibody goat anti-AXL (1:100, R&D Systems, AF854) overnight at 4°C followed by a secondary antibody Alexa Fluor 647 anti-goat (1:1500, Jackson,705 605 003) incubation for 2 h at RT. Afterwards, the rabbit anti-IBA1 (1:500, Wako, 019-19741) antibody was added for 48h at 4°C, followed by a secondary antibody Alexa Fluor 488 anti-rabbit (1:300, Invitrogen, A21206) incubation for 2 h at RT. Images of 10 serial coronal sections with 3 cortical regions each were taken with a Leica TCS SP5 confocal laser scanning microscope controlled by LAS AF scan software (Leica Microsystems, Wetzlar, Germany). The ImageJ software was used to process the stacks as followed: 16-bit conversion, Z Project with Maximum Intensity projection, despeckling, and measuring the Integrated Density of AXL, followed by analyzing IBA1 with enhancing the contrast with a saturation of 3 and setting the IsoData Threshold to 3 – 65535, converting it to a mask, removing outliers with a radius = 1 and a threshold = 100, the area was measured. As readout for changes in the DAM population the intensity of AXL was normalized to the respective area of IBA1.

For fluorescent co-labelling of BACE1 and 4G8, sections were blocked in 1x PBS with 10% Triton-X and 10% normal goat serum for 1 h at RT before adding the anti-BACE1 (1:500, Abcam, ab108394) antibody for 48 h at 4°C. After incubation with the secondary antibody Alexa Fluor 647 goat anti-rabbit (1:300, Invitrogen, A21244) for 1.5 h at RT, sections were blocked and incubated with the anti-4G8 (1:1000, Biolegend, SIG-39220-200) antibody for 24 h at 4°C. After incubation with the 568 donkey anti-mouse (1:500, Invitrogen, A10037) secondary antibody, cell nuclei were counterstained with DAPI (1:2000, Roche, 10236276001) and sections were embedded in fluorescent mounting medium (Dako, S3023). Images were taken as described above. Image processing, as well as data analysis was performed as described previously (1).

Western blot antibodies

| ACTIN | 1:10,000 | Sigma, A1978 |
| --- | --- | --- |
| APP | 1:2500 | Abcam, ab32136 |
| ARPC3 | 1:1000 | Proteintech, 14652-1-AP |
| ASC | 1:500 | Adipogene, AG-25B-0006-C100 |
| BACE-1 | 1:1000 | Abcam, ab108394 |
| BECN1 | 1:500 | Cell Signaling, 3495 |
| CTF | 1:500 | Sigma, A8717 |
| IDE | 1:1000 | Millipore, PC730 |
| LC3 | 1:500 | Sigma, L8918 |
| CASP1 and pro‐CASP1 | 1:500 | Abcam, ab179515 |
| IL‐1β and pro‐IL‐1β | 1:500 | eBioscience, 88701388 |
| NLRP3 | 1:500 | AdipoGen, AG‐20B‐0014 |
| NF-κB | 1:1000 | Cell Signaling, 4764s |
| pNF-κB | 1:1000 | Cell Signaling, 3039s |
| Gasdermin D | 1:500 | Adipogene, AG-25B-0036-C100 |

TaqMan primers

| *β-Actin* | ThermoFisher, Mm00607939_s1 |
| --- | --- |
| *Arpc3* | ThermoFisher, Mm07799871_m1 |
| *Casp1* | ThermoFisher, Mm00438023_m1 |
| *Cd36* | ThermoFisher, Mm01135198_m1 |
| *Ets2* | ThermoFisher, Mm00468973_m1 |
| *Hpgd* | ThermoFisher, Mm00515121_m1 |
| *Il-1β* | ThermoFisher, Mm00434228_m1 |
| *Il-6* | ThermoFisher, Mm00446190_m1 |
| *Nlrp3* | ThermoFisher, Mm00840904_m1 |
| *Plxna2* | ThermoFisher, Mm00801930_m1 |
| *Sirt3* | ThermoFisher, Mm00452131_m1 |
| *Tfeb* | ThermoFisher, Mm00448968_m1 |
| *Tnf-α* | ThermoFisher, Mm00443258_m1 |
| *Trem2* | ThermoFisher, Mm04209424_g1 |

Nuclei preparation for Single nuclei sequencing

Mouse hemispheres were harvested from male mice at the age of 180 days and immediately snap frozen in liquid nitrogen and stored at -80°C until further processing for nuclei isolation. Nuclei were isolated from a single mouse hemisphere in 2 ml of pre-chilled EZ PREP lysis buffer (NUC-101, Sigma) using a glass Dounce tissue grinder (D8938, Sigma) (25 strokes with pastel A and 25 strokes with pastel B) followed by incubation for 5 minutes on ice with additional 6 ml of EZ PREP buffer. During incubation, 1 µM DAPI was added to the homogenate and subsequently filtered through a 35 µm strainer. Intact nuclei were sorted with a BD FACSAriaIII with a 70 µm configuration into 1.5 ml-Eppendorf tubes with 40 µl of 4 % BSA in PBS and RiboLock RNase Inhibitor (25 U/µl, EO0381, ThermoFisher). A FSC/SSC based gate was used to exclude debris followed by exclusion of damaged nuclei in a DAPI-A/DAPI-H (see Supplementary Fig. 5a). 150,000 events were sorted for each sample. The concentration of sorted nuclei was determined based on brightfield images and DAPI fluorescence using a Neubauer counting chamber and a Leica DMi8 microscope.

Single nuclei sequencing

Single nuclei libraries were generated according to the Chromium Next GEM Single Cell 3ʹ Reagent Kits v3.1 User Guide (CG000204) by 10x Genomics. Briefly, a droplet emulsion was generated in a microfluidic chip followed by barcoded cDNA generation inside these droplets. Purified and amplified cDNA was then subjected to library preparation and sequenced on a NovaSeq 6000 instrument (Illumina) to a median depth of 45-90k reads per cell.

Single nuclei sequencing analysis

Sequencing libraries were processed with CellRanger (v3.1.0) against the mouse genome (mm10) augmented by intronic sequences and the APP and PS1 transgenes, followed by background removal with CellBender (2). snRNA-seq data analysis was performed in R (v3.6.3) with Seurat (v3.2.1) (3). Cells with at least 250 but less than 6000 genes and less than 10% mitochondrial RNA content were combined from each library, and clustering and UMAP embedding was computed based on log normalized gene counts, with total RNA content regressed out during scaling and using 20 PCA components. Cluster annotation was aided by marker expression and label transfer with Seurat's TransferData workflow using a previously published mouse brain dataset as reference (4). We compared cluster abundances between conditions using a mixed-effects binomial model and the lme4 package (v1.1-23). The cellular proportions (55% neurons and 45% glial cells) correlated well with proportions determined by the Blue Brain Cell Atlas in the cerebrum (59% neurons and 41% glial cells; (5)). Differential gene expression was performed using DESeq2 (6) on aggregated "pseudo-bulk" counts for each cluster from each sample. Pathway analysis was performed using tmod (7) (v0.44) and Gene Ontology terms from the msigdbr package (v7.2.1).

Sample preparation for proteomic analysis

Microglial cells isolated from mouse brain by MACS (approx. 800,000) were resuspended in 40 µl RIPA buffer with protease inhibitor and transferred to an AFA-TUBE TPX 8-Strip (Covaris, PN 520292, PN 500639). Proteins were extracted and DNA sheared in a Covaris LE220Rsc instrument with the following settings: Temperature 20°C, Duty Factor 25% and Plate Definition Dithering 1 mm, Y-dither @ 20 mm/s, peak power 350, duration 300 seconds, 2 repetitions. Cell debris were removed (2500 rpm for 5 min) and protein concentration determined (Pierce Protein Assay Kit, 23225). A volume corresponding to 25 µg protein was transferred and topped up to 50µl with RIPA buffer. Lysates were processed on a Biomek i7 workstation using the SP3 protocol as previously described with one-step reduction and alkylation (8). Briefly, 16.6 μl of reduction and alkylation buffer (40 mM TCEP, 160 mM CAA, 200mM ABC, 4% SDS) were added, samples were incubated at 95 °C for 5 min and cooled to RT. To bind the proteins, 250 μg of paramagnetic beads (1:1 ratio of hydrophilic/hydrophobic beads (GE Healthcare, PN 45152105050250, 65152105050250) were added and the proteins were precipitated by adding ACN to 50%. Samples were washed twice with 80% EtOH and once with 100% ACN before reconstitution in 35 μl 100 mM ABC. Samples were digested overnight at 37 °C (Benchmark Scientific Incu-Mixer MP4) with Trypsin/Lys-C mix (V5072) at a protein:enzyme ratio of 50:1 (w/w). The reaction was stopped by adding formic acid to a final concentration of 0.1%. Peptide concentration was determined (Pierce 23290), insoluble particles removed by centrifugation, samples transferred to a new plate and frozen at -80 °C until analysis by LC-MS/MS.

Liquid chromatography mass spectrometry

The tryptic digests were analysed on an UltiMate 3000 (Thermo Scientific Dionex) equipped with a Sonation column oven (PRSO-V1-BR, operated at 50C°) and coupled to a hybrid trapped ion mobility (TIMS) quadrupole time-of-flight instrument (timsTOF Pro2, Bruker Daltonics) operating in dia-PASEF mode. Peptides were loaded on a trap-column (PepMap C18, 5 mm × 300 μm × 5 μm, Thermo Scientific) at a flow of 20 μl/min over 5 min (2% ACN, 0.1% TFA in water) and separated on a 25 cm analytical column (Aurora series, CSI, 25 cm x 75 µm ID, 1.6 µm C18, IonOpticks) at 300 nl/min. The mobile phases were 0.1% FA in water (A) and ACN (B) respectively. Gradient elution was performed with the following program: increase from 2% B to 17% over 87 min, increase to 25% B over 6 min and 37% B in 3 min, followed by a wash with 80% B for 5 min, and equilibration with 2% B for 15 min (Thermo Scientific Price LC-MS grade). The nano-electrospray source (CaptiveSpray source, Bruker Daltonics) was operated at 1500 V of capillary voltage, 3.0 l/ min of drying gas and 180 C° drying temperature. For calibration of ion mobility dimension, three ions of Agilent ESI-Low Tuning Mix ions were selected (m/z [Th], 1/𝐾0 [Th]: 622.0289, 0.9848; 922.0097, 1.1895; 1221.9906, 1.3820). The dia-PASEF windows scheme was ranging in dimension m/z from 400 to 1200 and in dimension 1/𝐾0 0.7 – 1.43, with 32 x 25 Th windows with Ramp Time of 100 ms.

Proteomics data analysis

The raw data was processed using DIA-NN 1.8 (9) with the ion mobility module for diaPASEF (10). MS2 and MS1 mass accuracies were both set to 15 ppm, and scan window size was automatically optimised. DIA-NN was run in library-free mode with standard settings (fasta digest and deep learning-based spectra, RT and IMs prediction) using the Uniprot mouse reviewed (Swiss-Prot, downloaded on 2021-01-27) annotations (11) and the match-between-runs (MBR) option.

Peptide normalized intensities were subjected to quality control with all 16 samples passing acceptance criteria. Peptides with excessive missing values (> 34 % per group) were excluded from analysis. The missing values of remaining peptides were imputed group-based using the PCA method (12). Normalization was performed with LIMMA (13) implementation of cyclic loess method (14) with option “fast” (15). To obtain a quantitative protein data matrix, the log2-intensities of peptides were filtered, only peptides belonging to one protein group were kept, and then summarized into protein log intensity by “maxLFQ” method (16), implemented in R package iq (17).

Statistical analysis of proteomics data was carried out using internally developed R scripts based on publicly available packages. Linear modelling was based on the R package LIMMA (13). Following model was applied to each tissue data set (log(p) is log2 transformed expression of a protein): log(p) ~ 0 + Class. The categorical factor Class had four levels: APPPS1 H_2_O, APPPS1 spermidine, WT H_2_O, WT spermidine; reference level: APPPS1 H_2_O. Several contrasts were analyzed: Contrast1 = APPPS1 H_2_O – WT H_2_O (disease effect), Contrast2 = APPPS1 spermidine – APPPS1 H_2_O (spermidine effect in diseased species), Contrast3 = WT spermidine – WT H_2_O (spermidine effect in control species), Contrast4 = Contrast2 – Contrast3 (interaction of spermidine effect and disease effect), and Contrast5 = (Contrast2 – Contrast1)/2 (drug’s anti-disease effect).

For finding regulated features following criteria were applied: Significance level alpha was set to 0.04, which guaranteed for Contrast5 the Benjamini–Hochberg (18) false discovery rate below 30%. The log fold-change criterion was applied to guarantee that the measured signal is above the average noise level. As such we have taken the median residual standard deviation of linear model: log2 (T) = median residual SD of linear modelling (= log2(1.27)). Functional GSEA analysis was carried out using R package clusterProfiler (Yu et al., 2012). For selecting the most (de)regulated GO terms we applied filter: 3 ≤ term size ≤ 300.

The mass spectrometry proteomics data have been deposited to the ProteomeXchange Consortium via the PRIDE (19) partner repository with the dataset identifier PXD034638.

References

1. Eede P, Obst J, Benke E, Yvon-Durocher G, Richard BC, Gimber N, et al. Interleukin-12/23 deficiency differentially affects pathology in male and female Alzheimer's disease-like mice. EMBO Rep. 2020:e48530.

2. Fleming SJ, Marioni JC, Babadi M. CellBender remove-background: a deep generative model for unsupervised removal of background noise from scRNA-seq datasets. bioRxiv. 2019:791699.

3. Stuart T, Butler A, Hoffman P, Hafemeister C, Papalexi E, Mauck WM, et al. Comprehensive Integration of Single-Cell Data. Cell. 2019;177(7):1888-902.e21.

4. Schneeberger S, Kim SJ, Eede P, Boltengagen A, Braeuning C, Andreadou M, et al. The neuroinflammatory interleukin-12 signaling pathway drives Alzheimer’s disease-like pathology by perturbing oligodendrocyte survival and neuronal homeostasis. bioRxiv. 2021:2021.04.25.441313.

5. Erö C, Gewaltig M-O, Keller D, Markram H. A Cell Atlas for the Mouse Brain. Frontiers in Neuroinformatics. 2018;12.

6. Love MI, Huber W, Anders S. Moderated estimation of fold change and dispersion for RNA-seq data with DESeq2. Genome Biol. 2014;15(12):550.

7. Zyla J, Marczyk M, Domaszewska T, Kaufmann SHE, Polanska J, Weiner J. Gene set enrichment for reproducible science: comparison of CERNO and eight other algorithms. Bioinformatics. 2019;35(24):5146-54.

8. Müller T, Kalxdorf M, Longuespée R, Kazdal DN, Stenzinger A, Krijgsveld J. Automated sample preparation with SP3 for low-input clinical proteomics. Mol Syst Biol. 2020;16(1):e9111.

9. Demichev V, Messner CB, Vernardis SI, Lilley KS, Ralser M. DIA-NN: neural networks and interference correction enable deep proteome coverage in high throughput. Nat Methods. 2020;17(1):41-4.

10. Demichev V, Yu F, Teo GC, Szyrwiel L, Rosenberger GA, Decker J, et al. High sensitivity dia-PASEF proteomics with DIA-NN and FragPipe. bioRxiv. 2021:2021.03.08.434385.

11. UniProt: a worldwide hub of protein knowledge. Nucleic Acids Res. 2019;47(D1):D506-d15.

12. Josse J, Husson F. missMDA: A Package for Handling Missing Values in Multivariate Data Analysis. Journal of Statistical Software. 2016;70(1):1 - 31.

13. Ritchie ME, Phipson B, Wu D, Hu Y, Law CW, Shi W, et al. limma powers differential expression analyses for RNA-sequencing and microarray studies. Nucleic Acids Res. 2015;43(7):e47.

14. Bolstad BM, Irizarry RA, Astrand M, Speed TP. A comparison of normalization methods for high density oligonucleotide array data based on variance and bias. Bioinformatics. 2003;19(2):185-93.

15. Ballman KV, Grill DE, Oberg AL, Therneau TM. Faster cyclic loess: normalizing RNA arrays via linear models. Bioinformatics. 2004;20(16):2778-86.

16. Cox J, Hein MY, Luber CA, Paron I, Nagaraj N, Mann M. Accurate proteome-wide label-free quantification by delayed normalization and maximal peptide ratio extraction, termed MaxLFQ. Mol Cell Proteomics. 2014;13(9):2513-26.

17. Pham TV, Henneman AA, Jimenez CR. iq: an R package to estimate relative protein abundances from ion quantification in DIA-MS-based proteomics. Bioinformatics. 2020;36(8):2611-3.

18. Benjamini Y, Hochberg Y. Controlling the False Discovery Rate: A Practical and Powerful Approach to Multiple Testing. Journal of the Royal Statistical Society: Series B (Methodological). 1995;57(1):289-300.

19. Perez-Riverol Y, Bai J, Bandla C, García-Seisdedos D, Hewapathirana S, Kamatchinathan S, et al. The PRIDE database resources in 2022: a hub for mass spectrometry-based proteomics evidences. Nucleic Acids Res. 2022;50(D1):D543-d52.
